# The DEAH-box Helicase Dhr1 Dissociates U3 from the Pre-rRNA to Promote Formation of the Central Pseudoknot

**DOI:** 10.1371/journal.pbio.1002083

**Published:** 2015-02-24

**Authors:** Richa Sardana, Xin Liu, Sander Granneman, Jieyi Zhu, Michael Gill, Ophelia Papoulas, Edward M. Marcotte, David Tollervey, Carl C. Correll, Arlen W. Johnson

**Affiliations:** 1 Department of Molecular Biosciences and the Institute for Cellular and Molecular Biology, The University of Texas at Austin, Austin, Texas, United States of America; 2 Department of Biochemistry and Molecular Biology, Rosalind Franklin University of Medicine & Science, North Chicago, Illinois, United States of America; 3 Wellcome Trust Centre for Cell Biology, University of Edinburgh, Edinburgh, Scotland, United Kingdom; University of California, Berkeley, UNITED STATES

## Abstract

In eukaryotes, the highly conserved U3 small nucleolar RNA (snoRNA) base-pairs to multiple sites in the pre-ribosomal RNA (pre-rRNA) to promote early cleavage and folding events. Binding of the U3 box A region to the pre-rRNA is mutually exclusive with folding of the central pseudoknot (CPK), a universally conserved rRNA structure of the small ribosomal subunit essential for protein synthesis. Here, we report that the DEAH-box helicase Dhr1 (Ecm16) is responsible for displacing U3. An active site mutant of Dhr1 blocked release of U3 from the pre-ribosome, thereby trapping a pre-40S particle. This particle had not yet achieved its mature structure because it contained U3, pre-rRNA, and a number of early-acting ribosome synthesis factors but noticeably lacked ribosomal proteins (r-proteins) that surround the CPK. Dhr1 was cross-linked *in vivo* to the pre-rRNA and to U3 sequences flanking regions that base-pair to the pre-rRNA including those that form the CPK. Point mutations in the box A region of U3 suppressed a cold-sensitive mutation of Dhr1, strongly indicating that U3 is an *in vivo* substrate of Dhr1. To support the conclusions derived from *in vivo* analysis we showed that Dhr1 unwinds U3-18S duplexes *in vitro* by using a mechanism reminiscent of DEAD box proteins.

## Abstract 2

U3 snoRNA binds to pre-rRNA, helping to orchestrate key steps in ribosome assembly. This study identifies Dhr1 as the essential RNA helicase that releases U3 snoRNA and allows ribosome maturation to continue.

## Author Summary

Ribosomes are intricate assemblies of RNA and protein that are responsible for decoding a cell’s genetic information. Their assembly is a very rapid and dynamic process, requiring many ancillary factors in eukaryotic cells. One critical factor is the U3 snoRNA, which binds to the immature ribosomal RNA to direct early processing and folding of the RNA of the small subunit. Although U3 is essential to promote assembly, it must be actively removed to allow completion of RNA folding. Such RNA dynamics are often driven by RNA helicases, and here we use a broad range of experimental approaches to identify the RNA helicase Dhr1 as the enzyme responsible for removing U3 in yeast. A combination of techniques allows us to assess what goes wrong when Dhr1 is mutated, which parts of the RNA molecules the enzyme binds to, and how Dhr1 unwinds its substrates.

## Introduction

Ribosome biogenesis is fundamental to cellular growth. In bacteria that have undergone extreme genome reduction, ribosomes are apparently assembled without the use of specialized assembly factors [1], indicating that the information needed for the correct rRNA folding and protein assembly is intrinsic to the ribosomal components themselves. Similarly, functional bacterial ribosomes can be assembled from purified components *in vitro* [2,3]. Despite their general conservation of structure, eukaryotic ribosomes require a large number of protein and RNA trans-acting factors that assist in their assembly [4,5]. A central outstanding question in the field is how RNA-RNA and RNA-protein structural rearrangements, which mark the transition from one step to the next, are directed and regulated.

**Fig 1:**
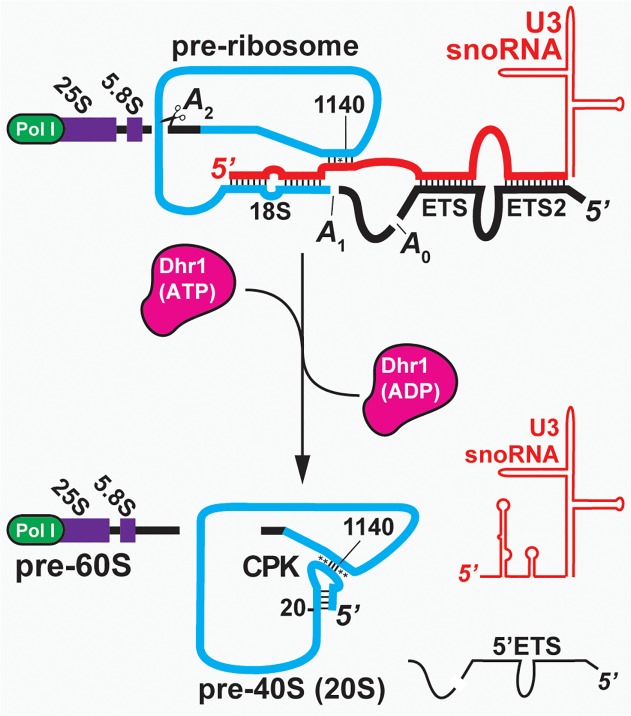
The transition from the pre-ribosome to the pre-40S. U3 binds to the pre-rRNA cotranscriptionally. Binding to the 5′-end of 18S and the downstream element (nts 1139–1142) brings together the elements of the CPK. Following cleavage at A0 and A1, U3 is displaced to allow folding of the CPK and cleavage at A2 to liberate the pre-40S. Watson-Crick base pairs (|), non-Watson base pairs (*).

Pre-ribosomal particles initially assemble on the nascent pre-ribosomal RNA (pre-rRNA) transcript, which undergoes cleavage to separate the pre-40S and pre-60S complexes. This critical event in ribosome biogenesis requires the U3 small nucleolar RNA (snoRNA). U3 is highly conserved among eukaryotes and base-pairs with multiple sites of the pre-rRNA to coordinate early folding and cleavage events [6–10]. The U3-associated proteins Imp3 and Imp4 promote the U3-pre-rRNA interactions *in vitro* [11–13], and are thought to serve a similar role *in vivo*. U3 binding to the 5′-external transcribed spacer (5′-ETS) and 18S regions of the pre-rRNA is required for the cleavage events at sites A0 within the 5′-ETS, at A1 that generates the mature 5′ end of 18S and at site A2, within internal transcribed spacer 1 (ITS1), which separates the earliest pre-40S and pre-60S particles [14,15]. Within the 18S rRNA, U3 binds to the sequence close to the 5′ end of the 18S rRNA that will form the 5′ side of the central pseudoknot (CPK), a long range interaction that is a key architectural feature of the small ribosomal subunit (SSU) in all domains of life [15]. U3 also has the potential to base-pair to the sequence that will form the 3′ side of the CPK and is located more than 1 Kb away in the 18S rRNA (Fig. 1), although this interaction has not been experimentally verified. These U3 interactions are believed to both facilitate formation of the CPK and control the timing of this key maturation step. However, U3 must be unwound from the pre-rRNA for CPK formation to occur. Furthermore, *in vivo* and *in vitro* studies indicate that the spontaneous dissociation rate of U3–18S interactions in the absence of accessory factors is too slow to support the rates of ribosome assembly observed *in vivo* [12,13,16], suggesting that a helicase is needed.

Nineteen RNA helicases are involved in ribosome biogenesis in yeast, 17 of which are essential [17–19]. These helicases are classified as either DEAD or DEAH/RHA enzymes based on conserved sequence motifs. DEAD box proteins do not unwind duplexes in a processive fashion. Rather, ATP-dependent binding to short duplex regions results in duplex destabilization and strand separation. Thus, ATP hydrolysis is not needed for duplex unwinding, but it is required for rapid product release to recycle the enzyme for multiple substrate turnovers. Processivity has also not been observed in DEAH/RHA enzymes but they have been less studied in mechanistic detail [20,21]. Identifying *in vivo* substrates for the RNA helicases has generally been challenging, and specific substrates have not yet been identified for most of the pre-ribosomal helicases. Previous analyses suggested two candidate helicases for the removal of U3 snoRNA from the CPK region. The DEAH helicase Dhr1 (Ecm16) was reported to be associated with U3 [22], whereas depletion of the DEAD enzyme Has1 leads to retention of snoRNAs, including U3, in pre-ribosomal particles [23]. Here, we provide genetic, *in vivo* cross-linking and biochemical evidence that Dhr1 is the helicase that directly displaces U3 from the pre-rRNA to permit formation of the CPK.

## Results

### Dhr1_K420A_ Accumulates a Novel ∼45S Particle Containing SSU Components

In a previous analysis of RNA helicases involved in SSU biogenesis in yeast, conserved motifs were systematically mutated to generate mutants defective in ATP binding and/or hydrolysis [18]. Over-expression of Dhr1 with a Lys420 to Ala mutation (Dhr1_K420A_) in the Walker A motif gave a dominant negative lethal phenotype, and inhibited pre-rRNA processing, primarily at sites A1 and A2 [18]. The dominant negative phenotype implies that this mutant efficiently competes with wild-type (WT) protein, possibly by binding unproductively to its substrate, but no specific RNA substrate was identified. We hypothesized that Dhr1 is involved in dissociation of U3 from the pre-rRNA and that the Dhr1_K420A_ mutant might block dissociation of the U3 complex.

To test this hypothesis, we ectopically expressed c-myc epitope-tagged, WT Dhr1 and the Dhr1_K420A_ mutant proteins in cells in which genomic *DHR1* gene was under control of the *GAL1* promoter and could be rapidly depleted by growth in glucose. This *P*_*GAL1*_ HA-*DHR1* strain was unable to grow on glucose-containing medium (S1A Fig.) and HA-tagged Dhr1 was depleted to levels below detection within 6 h of repression by glucose (S1B Fig.). *DHR1*–13myc fully complemented loss of *DHR1*, whereas *dhr1*_*K420A*_-13myc was unable to support growth (S2A Fig.).

**Fig 2:**
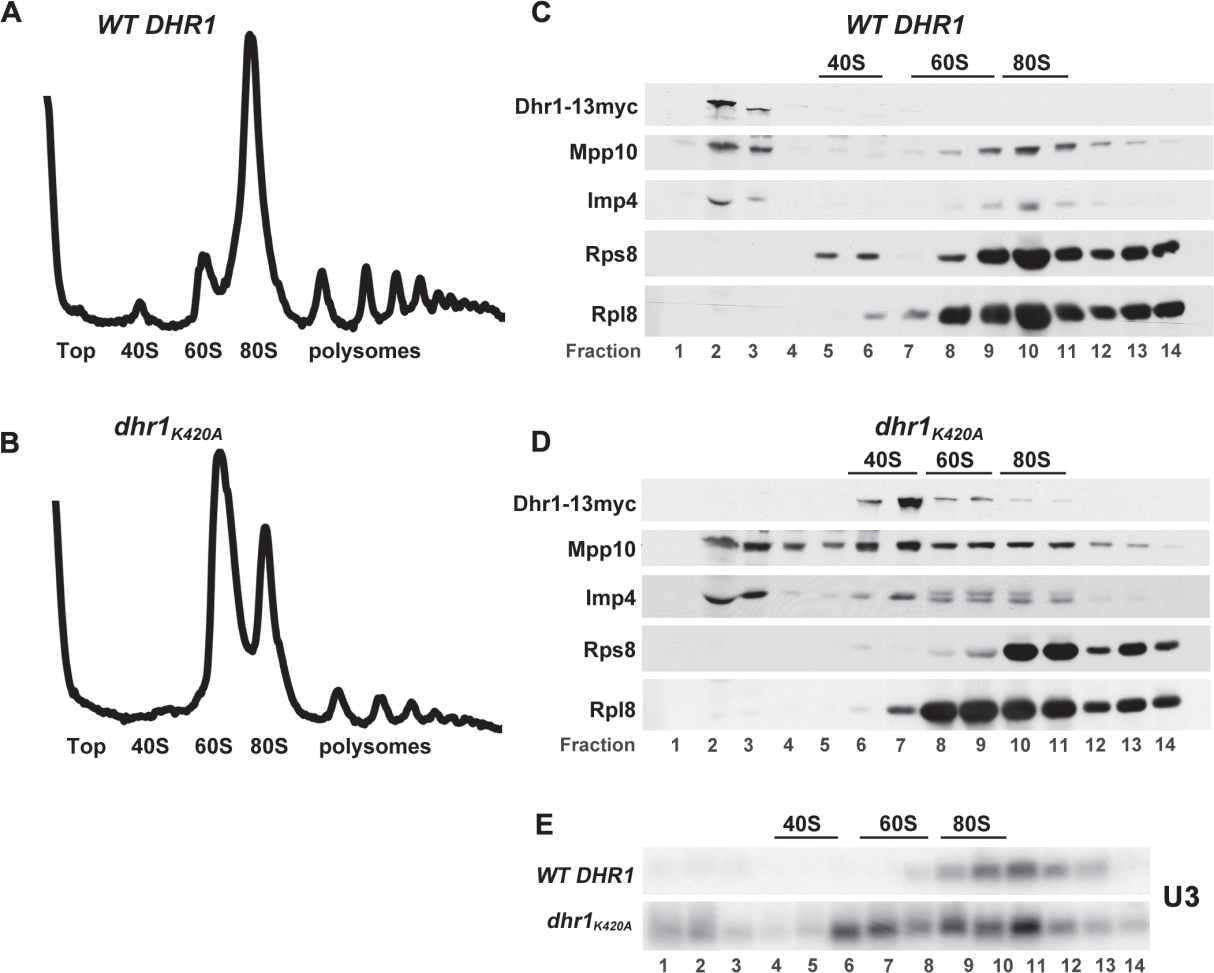
Dhr1_K420A_ accumulates a novel ∼45S particle containing SSU components. (A–D) Whole cell extracts were prepared from cycloheximide-treated cells expressing WT Dhr1–13myc (pAJ2311) (upper panels) or *dhr1-K420A* (pAJ3081) (lower panels) in yeast strain AJY3711 (*P*_*GAL*_-*3xHA-DHR1*) shifted to glucose media for 6 h to deplete genomically expressed 3xHA-Dhr1. (A, B) Extracts were subjected to sucrose density gradient ultracentrifugation and absorbance at 254 nm was monitored continuously throughout the gradients. Additional supporting data are provided in S1 Data. (C, D) Proteins were precipitated from fractions and subjected to SDS-PAGE followed by Western blotting. Dhr1–13myc, Mpp10, Imp4, Rps8, and Rpl8 were detected using anti-myc, anti-Mpp10, anti-Imp4, anti-Rps8, and anti-Rpl8 antibodies, respectively. The positions of 40S, 60S, and 80S, determined by monitoring absorbance at 254 nm, are indicated. (E) RNA was prepared from gradients as described for (A, B) and separated by agarose/formaldehyde gel electrophoresis. U3 was detected by Northern blotting using oligo AJO1686.

To identify the function of Dhr1, extracts from cells expressing Dhr1–13myc or Dhr1_K420A_-13myc were fractionated by sedimentation through sucrose density gradients. A strong 40S biogenesis defect in the mutant polysomes profile was evident from the loss of free 40S subunits and reciprocal increase in free 60S subunits in the *dhr1*_*K420A*_ mutant compared to WT (Fig. 2A and 2B). This reduction in free 40S was also evident from loss of Rps8 (eS8) from the 40S fraction in the *dhr1*_*K420A*_ gradient compared to WT (Fig. 2C and 2D). Western blotting for Dhr1 revealed that WT Dhr1 was almost entirely at the top of the gradient (Fig. 2C, lanes 2 and 3), indicating that the interaction of Dhr1 with pre-ribosomes is either very transient or unstable following cell lysis. In contrast, Dhr1_K420A_ sedimented at ∼45S (Fig. 2D, lane 7), indicative of stable association with pre-40S particles. The sedimentation at ∼45S was unexpected because Dhr1 was previously characterized as a factor that acts in the context of the 90S processome [15]. The altered Dhr1 sedimentation was accompanied by changes in the sedimentation of U3 (Fig. 2E, lanes 8–10 in WT and lane 6 in mutant) and its associated proteins Mpp10 and Imp4, with a significant fraction of these small nucleolar ribonucleoprotein (snoRNP) components co-sedimenting with Dhr1_K420A_ (compare Fig. 2C lanes 9–11 with Fig. 2D, lane 7).

**Fig 3:**
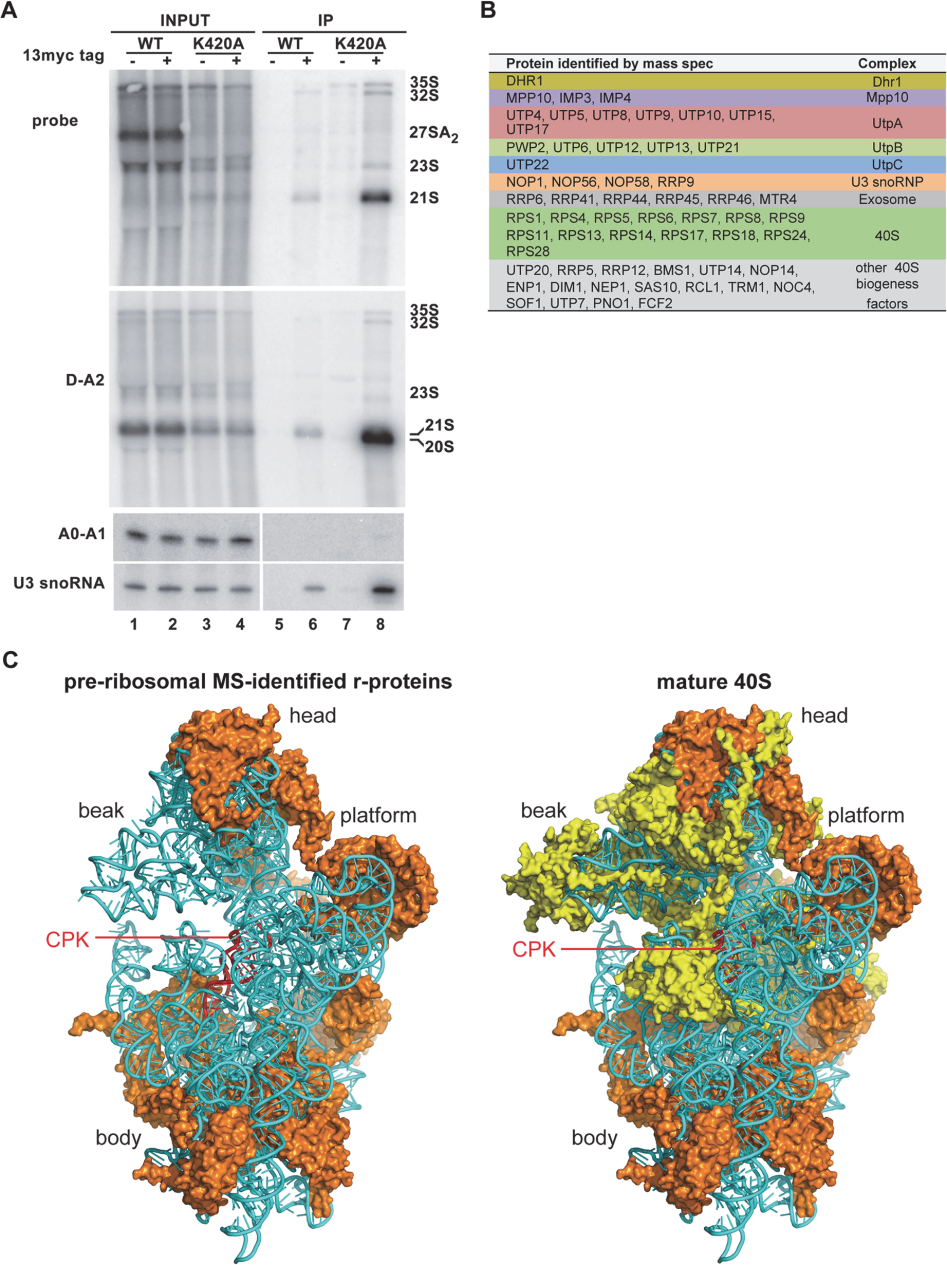
Dhr1_K420A_ co-immunoprecipitates U3 and associated proteins and 20S and 21S pre-rRNAs. (A) Cultures of AJY3711 (*P*_*GAL*_-*3xHA-DHR1*) expressing untagged WT Dhr1 (pAJ3082), WT Dhr1–13myc (pAJ2311), or *dhr1*_*K420A*_-*13myc* (pAJ3081) were shifted to glucose media for 6 h to deplete 3xHA-Dhr1. RNA was prepared from whole cell extracts (Input) or immunoprecipitated samples (IP) and separated by electrophoresis through agarose/formaldehyde gels or denaturing polyacrylamide gels for the A0-A1 fragment and U3. RNAs were detected by Northern blotting using probes specific to A2-A3 (AJO603), D-A2 (AJO130), A0-A1 (AJO1850), and U3 (AJO1686). (B) Table of proteins identified by MS in the Dhr1_K420A_ particle. Only proteins with at least three peptide-spectrum matches are listed. (C) The CPK (red), 18S rRNA (cyan), and r-proteins identified in the Dhr1_K420A_ particle (3B) are shown in orange in the structure of the mature *S*. *cerevisiae* 40S subunit (left). For comparison the proteins missing from the Dhr1_K420A_ particle are shown in yellow on the right. The Dhr1_K420A_ particle likely adopts a more open conformation in the absence of r-proteins.

Comparison of pre-rRNAs present in the strains expressing Dhr1 and Dhr1_K420A_ (Fig. 3A, lanes 1–4) revealed the loss of the 27SA2 pre-rRNA, accompanied by accumulation of an aberrant 21S species. The 5′ end of 27SA2 is generated by cleavage at site A2, whereas 21S is generated by cleavage at sites A1 and A3 in the absence of A2 cleavage. Notably, there was little accumulation of the 23S RNA, which is generated by A3 cleavage in the absence of cleavage at sites A0, A1, and A2, and is commonly seen in 40S subunit biogenesis mutants. These findings show that expression of Dhr1_K420A_ specifically impairs pre-rRNA cleavage at site A2. A reduced level of 20S pre-rRNA was detected in the mutant (Fig. 3A, lanes 3 and 4), showing that inhibition of A2 cleavage was not complete.

Pre-RNA species present in particles associated with Dhr1–13myc and Dhr1_K420A_-13myc were compared by immunoprecipitation (Fig. 3A, lanes 5–8). The mutant particle contained low levels of 35S and 32S pre-rRNAs, and was enriched for 21S and 20S pre-rRNAs (Fig. 3A). 21S RNA represented 36% and 20S represented the remaining 64% of the combined 21S + 20S signal.

We used mass spectrometry (MS) for a comprehensive analysis of the protein composition of the Dhr1_K420A_ particle (Fig. 3B; S1 Table). Epitope tagged (13xmyc) and untagged Dhr1_K420A_ particles were immunoprecipitated, digested with trypsin, and subjected to MS. The U3 snoRNA is specifically associated with the Mpp10 complex (Mpp10, Imp3, and Imp4) and Rrp9, as well the common box C/D snoRNA binding proteins Nop1, Nop56, Nop58, and Snu13. All were detected with the exception of Snu13, which is very small. Among the early binding factors, independently assembled complexes have been defined and termed the UtpA, B, and C complexes and Rrp5. The MS analysis detected seven of the eight subunits of Utp-A complex, all six components of the Utp-B complex, two Utp-C components, Rrp5, and 19 of the 33 SSU ribosomal proteins (r-proteins). Notably absent were the late assembling r-proteins, including Rps2 (uS5), Rps3 (uS3), and Rps23 (uS12). The absence of Rps2, Rps3, and enrichment of Imp4 and Mpp10 were confirmed by Western blotting (S3 Fig.). Numerous additional 40S biogenesis factors, including the GTPase Bms1 and the putative A2 endonuclease Rcl1 [24] were also present, but late biogenesis factors, such as Ltv1, were not detected. Surprisingly, nine of the 11 subunits of the nuclear exosome were identified together with its cofactor, the RNA helicase Mtr4 [25]. The presence of the nuclear exosome could reflect its activity in removing the cleaved fragments of the 5′-ETS or recognition of the particle as defective by the nuclear surveillance machinery. The protein and RNA composition of this particle indicates that it is arrested at an early stage in pre-40S assembly, in which U3 and the SSU-processome complex remain associated with the pre-rRNA [26].

Mapping the r-proteins present in the Dhr1_K420A_ particle to the mature 40S structure revealed a remarkable absence of r-proteins surrounding the CPK (Fig. 3C). In particular, Rps2, the primary r-protein that binds to the CPK, and Rps23 were absent from this particle. Comparison with the bacterial *in vivo* assembly map [27] revealed that almost all of the primary and secondary binding r-proteins were present but tertiary binding proteins were absent. We propose that the absence of proteins surrounding the CPK allows flexibility in the ribosomal RNA structure to enable access of assembly factors, including Dhr1 and the U3 snoRNA, to the CPK.

### U3 Remains Base-Paired with 18S in a *dhr1* Mutant

**Fig 4:**
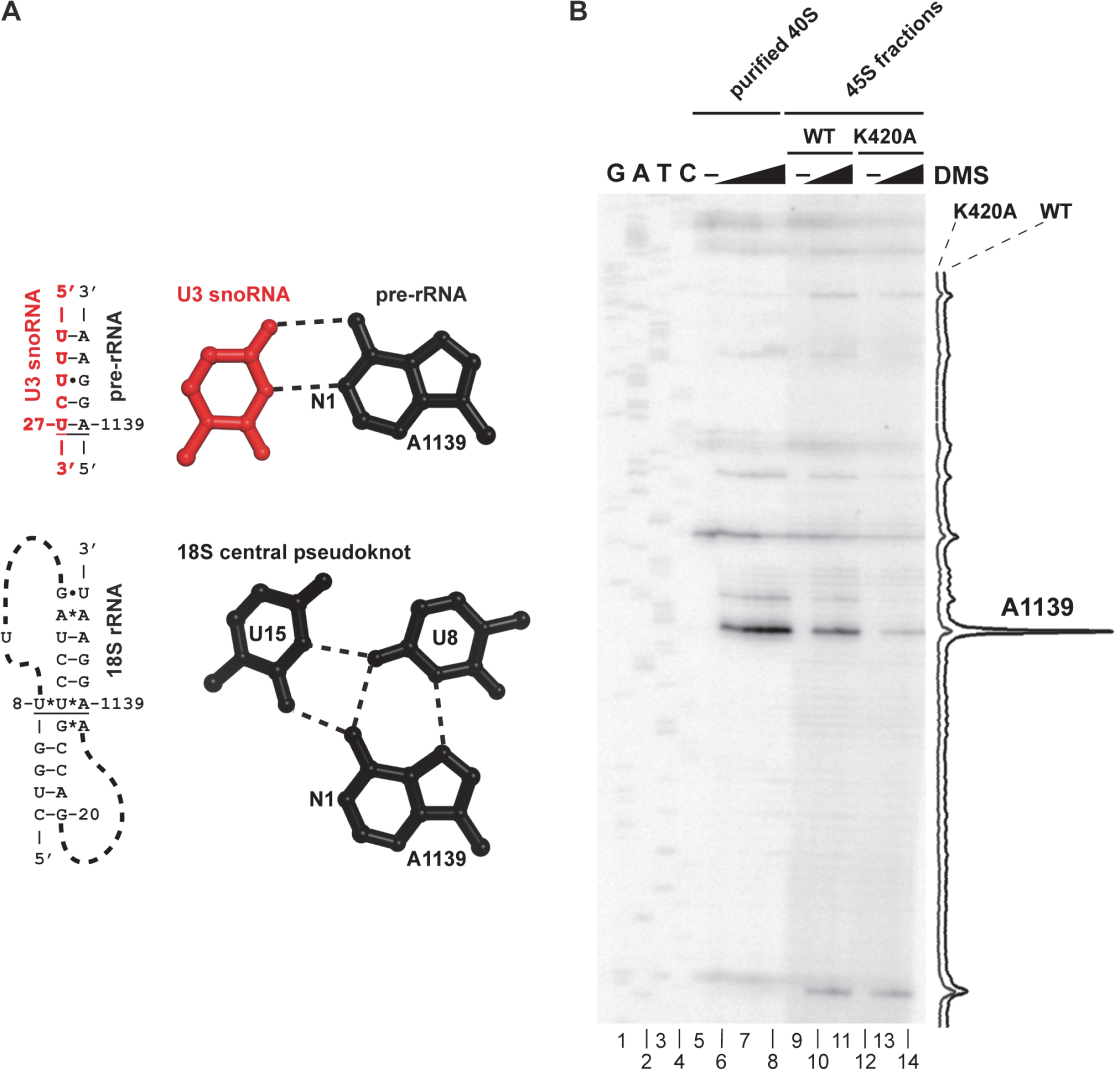
U3 remains base-paired with 18S rRNA in a *dhr1* mutant. (A) Cartoon showing expected U3–18S rRNA base-pairing and the same region of 18S rRNA in the CPK of the mature 40S subunit, based on crystal structure (PDB 3U5B). (B) DMS was used to probe accessibility of A1139. Extracts were prepared from cells expressing WT Dhr1 or Dhr1_K420A_, as described in the legend to Fig. 2 and fractionated on sucrose density gradients. The 45S regions of the gradients were harvested and treated with DMS as indicated. DMS modification was detected by primer extension using radiolabeled primer AJO1849. Peak areas were quantified using ImageJ (NIH). Purified mature 40S subunits were used as a control and a sequencing ladder was generated using a DNA template containing the 18S rDNA gene.

If Dhr1 is responsible for displacing the U3 from the pre-18S, then U3 should remain base-paired with 18S in the ∼45S particle that accumulates in Dhr1_K420A_ expressing cells. To examine this possibility, we used chemical modification with dimethyl sulfate (DMS), which modifies N1 of adenines and N3 of cytosines, and primer extension. When the pre-18S is base-paired with U3, A1139 is predicted to be in a standard Watson-Crick A-U base pair and thus protected from DMS (Fig. 4A top) [7]. In contrast, in the mature CPK A1139 is involved in a non-Watson-Crick base triple [28] and N1 is susceptible to DMS modification (Fig. 4A, bottom) [29]. Thus, the DMS susceptibility of A1139 is diagnostic for whether the CPK has formed or remains base-paired with U3.

Extracts from cells expressing Dhr1 or Dhr1_K420A_ were fractionated by sucrose density gradient sedimentation, and fractions corresponding to ∼45S were collected. We anticipated that in WT cells this fraction would primarily contain mature 40S particles whereas in the mutant, the limited pool of 40S would rapidly recycle into translating ribosomes and the stalled intermediate containing Dhr1_K420A_ would accumulate. Pooled fractions were treated with DMS or mock and purified mature 40S subunits were treated for a control. Modification of A1139 was readily detected in mature 40S subunits (Fig. 4B, compare lane 5, no DMS, with lanes 6–8) and in 45S fractions from the WT gradient (Fig. 4B, lanes 9–11). However, A1139 modification was substantially reduced in the mutant (Fig. 4B, lanes 12–14). The particularly strong signal for A1139 in mature subunits probably reflects the hypersensitivity of this position to DMS, as reported for A915 of the bacterial ribosome (analogous to yeast A1139) [30]. The relative decrease of A1139 modification was quantified and normalized to intensities of nearby peaks that were relatively constant between WT and mutant (Fig. 4B, lane quantification, far right). Reactivity of A1139 to DMS was reduced 80% in the mutant compared to WT. This low reactivity indicates that A1139 remains base-paired with U3 in the Dhr1_K420A_ particle.

### Dhr1 Directly Binds U3 snoRNA Elements Required for Base-Pairing with Pre-rRNA

**Fig 5:**
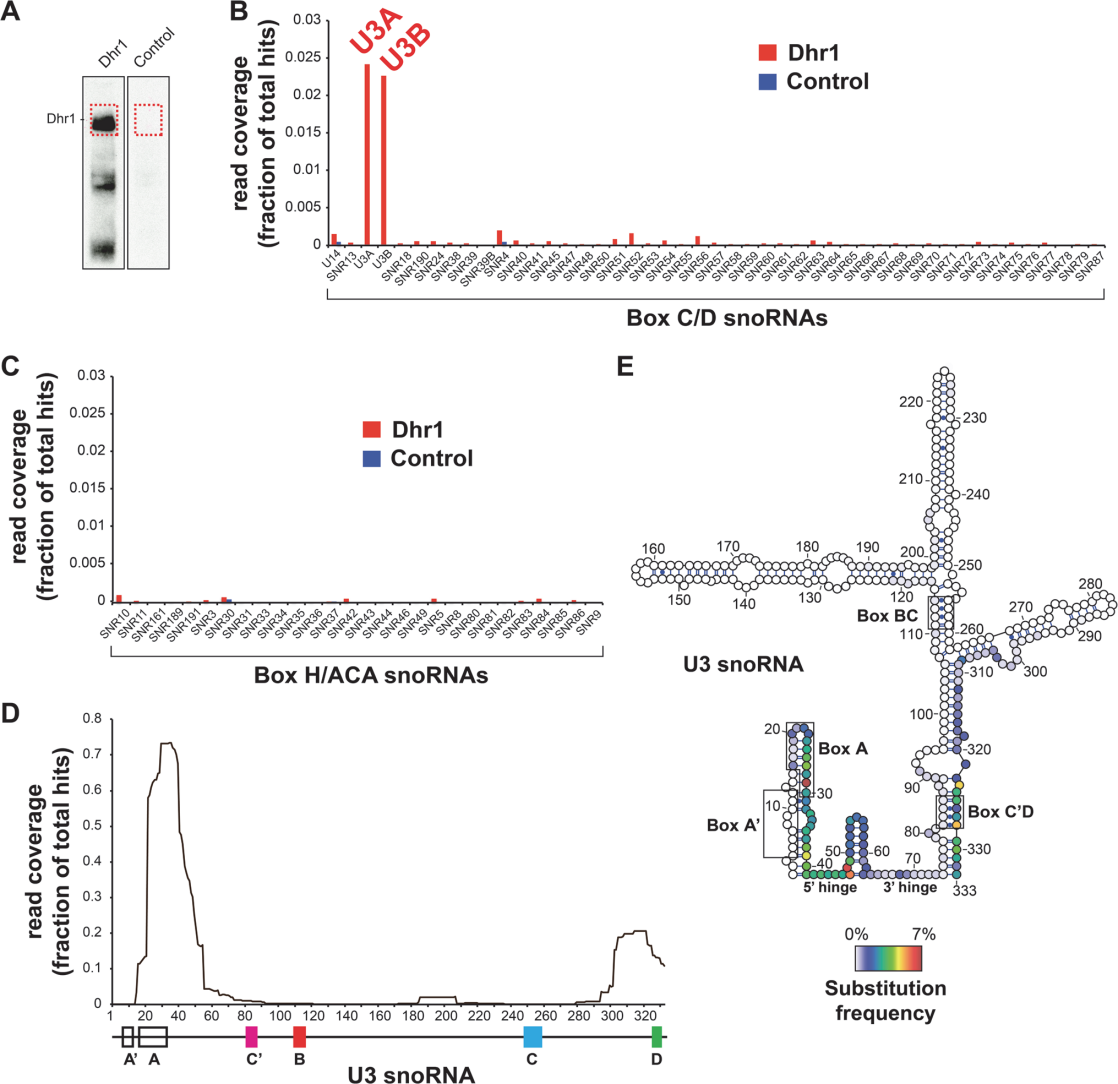
Dhr1 cross-links to U3. (A) The parental and a Dhr1-HTP tagged strain were subjected to the CRAC protocol (see Materials and Methods), cross-linked RNA was partially digested, radioactively labeled, ligated to linkers, and after nickel purification resolved on a 4%–12% NuPAGE gel. Protein-RNA complex was transferred to nitrocellulose and RNA was extracted from the regions indicated by a red dashed box. (B) Dhr1 preferentially cross-links to U3. Reads from Dhr1 (*n* = 2) and a negative control CRAC experiment were mapped to the 2008 *S*. *cerevisiae* genomic reference sequence and mapped reads were assigned to genomic features. The histogram shows the average percentage of all mapped reads that contained box C/D and (C) box H/ACA snoRNA sequences. Note that only a very small fraction of the reads from the control experiment mapped to snoRNAs. (D) Dhr1 preferentially cross-links to the 5′ end of the U3. Plotted is the average read distribution frequency over the U3A (snR17A) gene generated from two Dhr1 CRAC datasets. A schematic representation of the U3 gene and functional sequence elements are indicated below the plot. (E) Same as in (D) but for nucleotide substitutions. The secondary structure of the U3 was adopted from Granneman and colleagues [31] and generated using VARNA (http://varna.lri.fr). The coloring indicates the frequency by which the nucleotide was substituted. Additional supporting data are provided in S2 Data.

**Fig 6:**
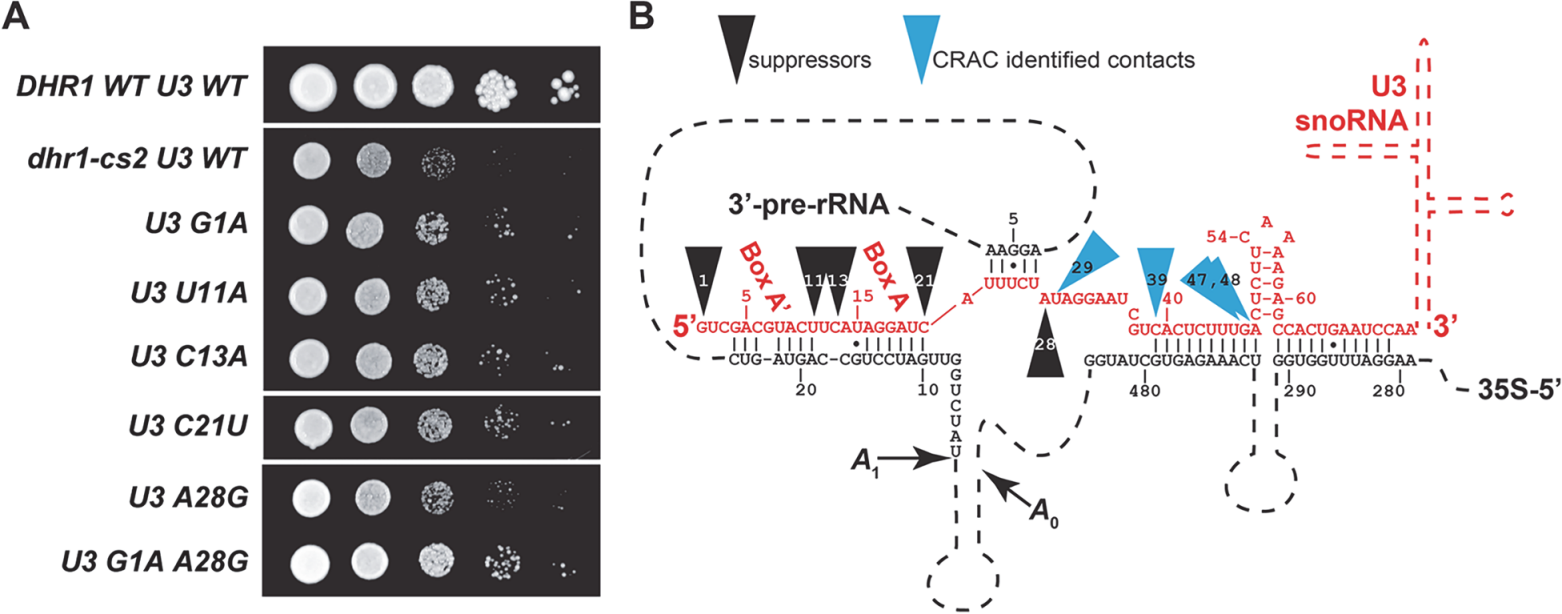
Mutations in the 5′-end of U3 suppress a cold-sensitive dhr1 mutant. (A) Random mutations were introduced the entire coding region of U3 (*SNR17A*) by low fidelity PCR. The mutant PCR library was then recombined into an expression vector *in vivo* in AJY3752 (*P*_*GAL*_-*DHR1 P*_*GAL*_-*SNR17A snr17B∆*) expressing *dhr1-cs2* (pAJ3095) and transformants were screened for improved growth at 20°C. Individual mutations from suppressing clones were subcloned and their ability to suppress *dhr1-cs2* was compared by serial dilution assay. Top row, WT *DHR1* with WT U3; second row, *dhr1-cs2* with WT U3 as controls. Plates were incubated at 20°C for 7 days. (B) The positions of U3 mutations that suppress *dhr1-cs2* and residues most frequently cross-linked to Dhr1, identified by substitutions in the CRAC analysis, are mapped to the predicted U3–18S secondary structure.

To identify direct RNA binding sites of Dhr1, we performed UV cross-linking and analysis of cDNA (CRAC) experiments on strains expressing Dhr1 with a tripartite C-terminal tag, consisting of His6—tobacco etch virus protease (TEV) cleavage site—protein A (Dhr1-HTP), and untagged Dhr1 as a negative control [31] (Fig. 5). UV cross-linking of Dhr1 *in vivo* yielded a strong RNA cross-linked species for Dhr1-HTP but not for the control (Fig. 5A). As other RNA helicases have been shown to be required for the release of snoRNAs from the pre-rRNA, we compared the read density of Dhr1 across all snoRNAs in yeast. The snoRNAs have been divided into two large groups termed box C/D and box H/ACA (Fig. 5B and 5C), on the basis of conserved sequence elements and common proteins. Dhr1 had a strong preference for cross-linking only to the box C/D U3 (encoded by U3A and U3B, Fig. 5B); no other snoRNA was significantly enriched. Within U3 most of the reads overlapped within the box A motif in the 5′ end of the RNA, with smaller numbers of hits at the 3′ terminal stem and box D (Fig. 5D). In CRAC analyses, the locations of micro deletions or substitutions in the cDNAs indicate the precise sites of protein-RNA cross-linking. These contact sites could be to the helicase active site itself or to other surfaces of the protein that bind to RNA. Deletions in the Dhr1 reads were rare but U29, C39, G47, and A48 were frequently substituted (Fig. 5E), indicating that Dhr1 directly contacts these nucleotides in U3. Notably, U29 is located in the box A motif directly downstream of the predicted U3 interaction with the 3′ side of the CPK while C39, G47, and A48 flank a U3 binding site for the 5′-ETS (Figs 5E and 6C).

We also investigated Dhr1 cross-links to pre-rRNA. Dhr1 cross-linking was detectable at many sites on pre-rRNA, but three peaks, in helices H11, H23, and H44 in the SSU, were reproducibly identified as strong cross-linking sites in two independent experiments (S4A and S5 Figs). We mapped these sites onto the 18S rRNA based on the yeast ribosome crystal structure [28]. The cross-linking sites are located near the decoding center of the SSU (at the conjunction of H44, H45, and H11) and in the platform area (S4B Fig.), consistent with Dhr1 playing a role in formation of the functional center of the 40S subunit. RNA isolated from a control CRAC experiment with the parental strain yielded mainly 25S rRNA sequences that are common contaminants in many CRAC experiments (S4C Fig.) [32]. Collectively, the CRAC analysis positions Dhr1 on the U3-snoRNA adjacent to the U3–18S duplex with the pre-rRNA.

### Mutations in U3 Suppress a Cold-Sensitive *dhr1* Mutant

We hypothesized that cold-sensitive mutations in Dhr1 might stall a reaction intermediate containing the U3–18S duplex. If this duplex is indeed the target of Dhr1, then mutations in U3 that destabilize the duplex might suppress the *dhr1* mutation. Cold-sensitive *dhr1* mutants were therefore identified by screening cells expressing randomly mutagenized *DHR1*. Yeast cells carrying *dhr1-cs2* were strongly cold-sensitive (S6A Fig.), displayed a strong 40S biogenesis defect (S6B Fig.), and, like the K420A mutant, accumulated U3 at the position of ∼45S in a sucrose density gradient (S6B Fig.). Sequencing *dhr1-cs2* revealed multiple mutations: E330G, K399E, T422A, A557G, T633A, and K892E. We then randomly mutagenized *SNR17A* (encoding U3A) by highly mutagenic PCR. PCR product was recombined into a U3 expression vector *in vivo* in a *dhr1-cs2* and conditional U3 mutant strain and transformants were screened for improved growth at low temperature. In cases where *SNR17A* contained multiple mutations, these were separated by subcloning. This screen identified five single point mutations that suppressed the cold-sensitivity of *dhr1-cs2* (Fig. 6A). Notably, all of the suppressing mutations mapped to the extreme 5′-end of U3, from residues G1 to A28 (Fig. 6B), despite the fact that the entire gene was mutagenized. A28G was the most frequently recovered mutation (present in seven of 12 suppressing mutants), with the double mutation of G1A/A28G showing the strongest growth suppression.

The position of these mutations was remarkably coincident with our CRAC data in which Dhr1 most strongly protected residues 22 to 55 of U3 immediately downstream of the suppressing mutations (Fig. 6B). Moreover, the residue most frequently substituted in cDNAs from our CRAC analysis was U29, adjacent to A28, the residue most commonly mutated in our suppressor screen. Such genetic suppression of a helicase mutant by mutations in an RNA provides strong evidence that the RNA is an *in vivo* target of the helicase.

### Dhr1 Has RNA-Dependent ATPase Activity

**Fig 7:**
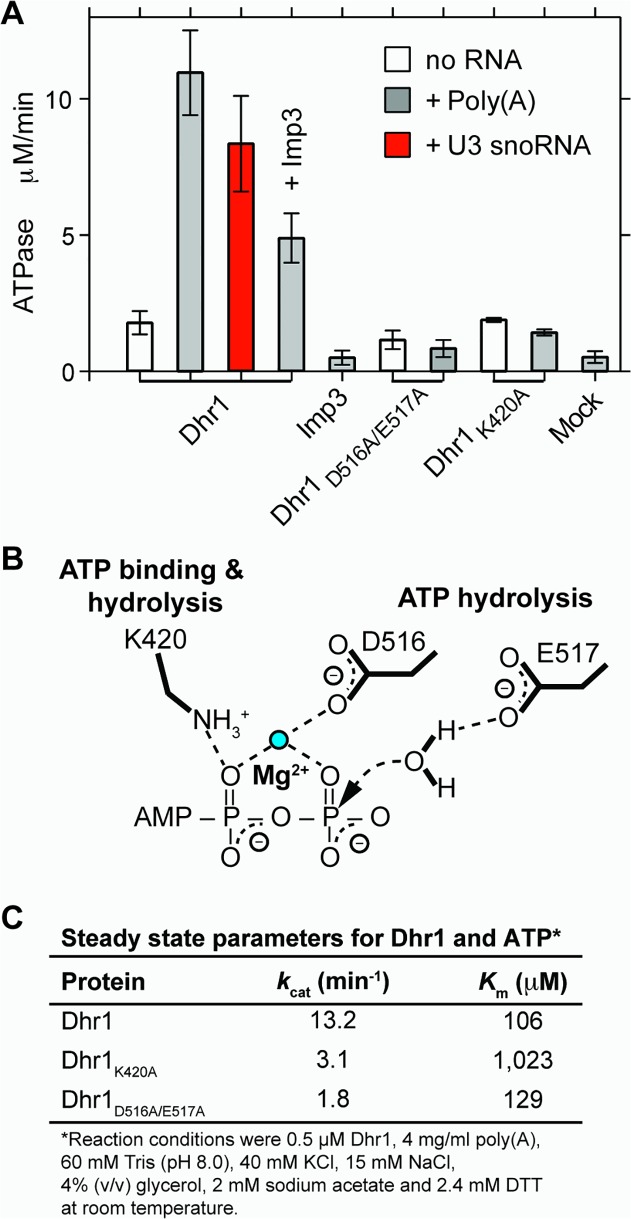
*In vitro* ATPase activity of Dhr1. (A) Initial velocities of P_i_ released after addition of 1 mM ATP at room temperature (RT) in the presence or absence of the indicated RNA with either WT Dhr1, Dhr1_D516A/E517A_, Dhr1_K420A_, or mock. Activity of Dhr1 either in the presence or absence of Imp3 was also determined. (B) Schematic of the putative ATP binding site of Dhr1 with expected contacts from K420 (motif I) and from D516 and E517 (motif II). (C) Determination of the ATP *k*_cat_ and *K*_m_ for Dhr1 with the Michaelis-Menten plots shown in S7 Fig. Additional supporting data are provided in S3 and S4 Data.

The above data indicated that Dhr1 directly dissociates U3 from the pre-rRNA *in vivo*. To test this activity *in vitro* we expressed Dhr1 with a C-terminal His6 tag in *Escherichia coli* and purified the recombinant protein. *DHR1*-His6 fully complemented a *dhr1* null mutant in yeast (S2B Fig.), verifying that the tag did not interfere with its function. We first investigated whether Dhr1 shows RNA-stimulated ATPase activity, a hallmark of DEAH/RHA RNA helicases. Dhr1 displayed weak ATPase activity in the absence of added RNA. Addition of single stranded poly(A) RNA or U3 snoRNA stimulated this activity 6-fold and 5-fold, respectively (Fig. 7A).

To confirm that the observed activities could be ascribed to Dhr1 and not a copurifying contaminant, we generated two active site mutants. The Walker A Dhr1_K420A_ mutant described above is expected to disrupt ATP binding and thus inhibit ATP hydrolysis because the side chain of this lysine is expected to contact the β phosphate of the ATP (Fig. 7B). In contrast, the Dhr1_D516A/E517A_ mutant is expected to disrupt ATP hydrolysis by removing the carboxylic acids in motif II (Walker B box) that bind the catalytic metal ion and activate the nucleophilic water molecule (Fig. 7B). RNA-dependent stimulation of ATPase activity was not observed in either mutant (Fig. 7A). Steady state kinetic parameters were determined (Figs 7C and S7 and Material and Methods) from the dependence of ATPase activity on input ATP concentration: *K*_m_ of 106 μM and *k*_cat_ of 13 min^−1^. As expected for a mutant whose main defect is in ATP binding, the *K*_m_ of Dhr1_K420A_ increased by an order of magnitude, from 106 μM to 1 mM, but *k*_cat_ decreased by only 4-fold, from 13 to 3 min^−1^. As expected for the catalytic mutant Dhr1_D516A/E517A_, the *K*_m_ of the Dhr1_D516A/E517A_ mutant remained almost unchanged, 106 μM versus 136 μM, whereas *k*_cat_ decreased by 7-fold, from 13 to 1.8 min^−1^.

### Dhr1 Unwinds a U3-ETS2 Duplex *In Vitro*

**Fig 8:**
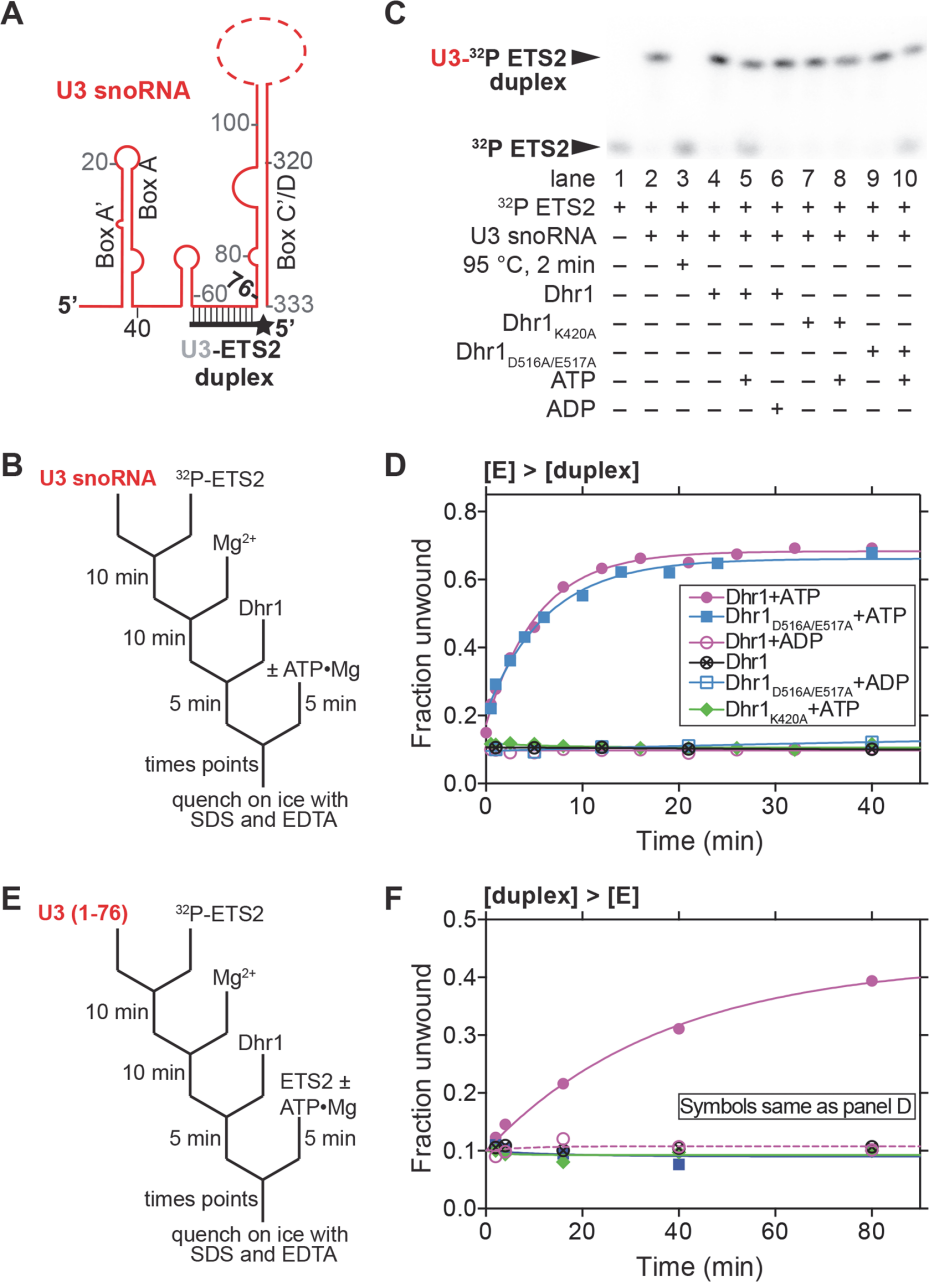
U3-ETS2 duplex unwinding by Dhr1. (A) Cartoon of U3-ETS2 substrate used for unwinding assays. Full length U3 snoRNA was used for the reactions in (B, C, and D). A truncated version of U3 snoRNA (nts 1–76) was used in the reactions in (E and F) to avoid non-specific interaction between Dhr1 and the 3′ region of U3 snoRNA. (B) Reaction scheme for the pre-steady state conditions ([E] > [duplex]) with results in (C and D). (C) Representative unwinding reactions stopped after 20 min. Electrophoretic mobility shift assay (EMSA) separated the ^32^P-labeled ETS2 free (unwound) from its duplex form. The RT reaction contained 500 nM Dhr1, 1 mM ATP, ≤0.3 nM U3-ET2 duplex with other reagents described in Materials and Methods. (D) Fraction unwound is plotted as a function of time: after addition of either ATP in the presence of either Dhr1 (purple circle), Dhr1_D516A/E517A_ (blue square), Dhr1_K420A_ (green diamond); after addition of ADP in the presence of either Dhr1 (open purple circle) or Dhr1_D516A/E517A_ (open blue square); or after addition of Dhr1 (circle with a cross). (E) Outline of the turnover of excess substrate reaction scheme under steady state conditions ([duplex] > [E]) with results in (F). The reaction contained 100 nM Dhr1, 6 mM ATP, 500 nM U3-ETS2 radiolabeled duplex, and 5 μM of unlabeled ETS2 with other reagents described in Materials and Methods. (F) Fraction unwound is plotted as a function of time with symbols as in (D). Additional supporting data are provided in S5 and S6 Data.

**Fig 9:**
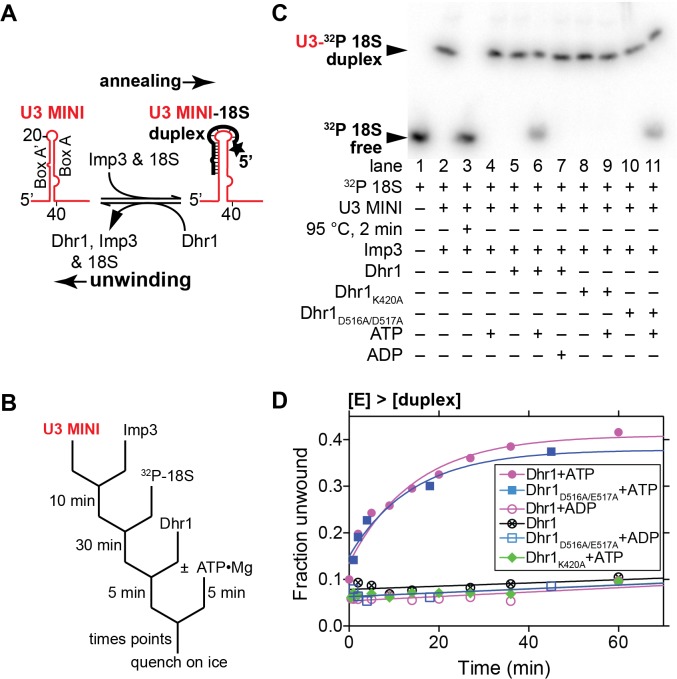
U3–18S duplex unwinding by Dhr1. (A) Reaction schematic for annealing the U3–18S duplex by Imp3 and duplex unwinding by Dhr1. (B) Reaction scheme for the pre-steady state conditions ([E] > [duplex]) with results shown in (C and D). (C) Representative RT unwinding single time point data after 60 min. From EMSA showing ^32^P-labeled 18S free (unwound) or in duplex with U3 MINI in a complex with Imp3 using 500 nM Dhr1, 1 mM ATP, ≤1 nM U3–18S duplex and other reagents described in Materials and Methods. (D) Time-dependent reactions with symbols as defined in Fig. 8D. Additional supporting data are provided in S7 Data.

We next tested the unwinding activity of Dhr1 on a U3-ETS2 duplex that mimics one of the three genetically verified U3-pre-rRNA duplexes (Figs 8 and 9) [11–13]. The U3-ETS2 duplex comprises the 3′ hinge of U3 bound to nts 281 to 291 of the 5′-ETS of the pre-rRNA (Fig. 8A). This duplex is required for subsequent U3-pre-rRNA interactions *in vivo* [7] and forms spontaneously and is stable *in vitro* [13]. U3-ETS2 duplex unwinding reactions were performed under pre-steady state conditions with an excess of enzyme over the duplex substrate. In addition, the duplex concentration was limiting to minimize duplex reformation after unwinding. Under these conditions, the U3-ETS2 duplex was efficiently unwound by Dhr1 in the presence of ATP but not in its absence or in the presence of ADP when the reaction was monitored at a single (Fig. 8C, compare lanes 4, 5, and 6) or at multiple time points (Fig. 8D). These data illustrate that duplex unwinding is ATP dependent.

To examine whether ATP hydrolysis is required for unwinding, we carried out reactions under the pre-steady state conditions described above using the ATP binding mutant Dhr1_K420A_ and the catalytically impaired mutant Dhr1_D516A/E517A_. In the presence of ATP, unwinding activity of Dhr1_D516A/E517A_ was indistinguishable from that of WT Dhr1 (Fig. 8C, compare lanes 5 and 10, and Fig. 8D). In contrast, no unwinding activity was observed for Dhr1_K420A_ in the presence of ATP or with Dhr1_D516A/E517A_ in the presence of ADP. These results indicate that ATP binding but not hydrolysis is needed for duplex unwinding activity.

To test whether product release requires ATP hydrolysis we measured duplex unwinding activity under steady state conditions in which duplex substrate was present in excess over enzyme. To circumvent the problem of the labeled substrate re-annealing, we used excess unlabeled ETS2 in a strand exchange regime [33]. Under these conditions, repeated release of unwound product by Dhr1 is necessary for efficient unwinding. Unwinding by WT Dhr1 was observed under steady state conditions in the presence of ATP. In contrast, neither mutant was active under these conditions in the presence of ATP (Fig. 8F). Thus, two lines of evidence indicate that ATP hydrolysis by Dhr1 is required for product release from the enzyme but not for duplex unwinding. First, the catalytically impaired Dhr1_D516A/E517A_ supported unwinding activity in the presence of ATP under pre-steady state but not steady state conditions. Second, Dhr1_K420A_, which is impaired in ATP binding, was inactive under both conditions. This requirement of ATP hydrolysis by Dhr1 for efficient enzyme recycling but not duplex unwinding suggests that Dhr1 shares a common mechanism with DEAD box proteins [34].

### Dhr1 Unwinds a U3–18S Duplex *In Vitro*

We also tested the ability of Dhr1 to unwind a second duplex, U3–18S, composed of box A’/A of U3 hybridized to nts 6–22 of 18S (Fig. 9A). We previously developed an *in vitro* assay for formation of the U3–18S duplex [11–13] using a minimal system containing nts 4–50 of U3, designated U3 MINI, and nts 6–22 of 18S rRNA to mimic the U3-18S duplex (Fig. 9A). This duplex does not form spontaneously but forms rapidly in the presence of Imp3, which unfolds stem-loop structures in the U3 and pre-rRNA to expose the sites of hybridization [12,13]. Our genetic results indicate that the U3–18S duplex mimics a *bona fide* Dhr1 substrate.

As was observed with the U3-ETS2 duplex, unwinding of the U3–18S duplex was observed in the presence of ATP under pre-steady state conditions, but was not observed either in the absence of ATP or in the presence of ADP (Fig. 9C, lanes 5–7, and Fig. 9D). Moreover, unwinding activity was observed for the Dhr1_D516A/E517A_ mutant in the presence of ATP, but not for Dhr1_K420A_ with ATP (Fig. 9D). These data suggest that unwinding requires ATP binding but is independent of ATP hydrolysis. Although we expect that rapid product release from the U3–18S substrate requires ATP hydrolysis, we were unable to observe unwinding by Dhr1 under steady state conditions with this substrate. The extent of unwinding of the U3–18S duplex by Dhr1 was less than that observed for the U3-ETS2 duplex reaction, presumably because unwinding by Dhr1 competes with strand annealing promoted by Imp3, which is present in excess. Imp3 does not appear to contribute to duplex unwinding, irrespective of the presence of ATP in the reaction (Fig. 9C, lane 4). Moreover, Imp3 binding decreased the RNA-dependent ATP hydrolysis by Dhr1 (Fig. 7A). Because the presence of Imp3 is necessary to maintain the U3–18S duplex [11], we have not been able to separate RNA unwinding from RNP remodeling activity with this substrate. Thus, it is currently unclear whether the activity of Dhr1 removes Imp3 directly, destabilizes the U3–18S duplex, or both.

## Discussion

RNA helicases are required for many steps in RNA metabolism, including splicing, RNP assembly, translation, and RNA degradation. However, there are few cases in which clear genetic relationships between substrate and helicase or *in vitro* assays with a relevant substrate have been established. Here, we have combined genetic, CRAC, and biochemical evidence to conclude that Dhr1 is the helicase that displaces U3 from the 5′ end of the 18S RNA portion of the pre-rRNA to allow formation of the CPK. Our data show that mutant Dhr1 traps a preribosome particle in which U3 remains base-paired with 18S rRNA. Dhr1 cross-links to U3 immediately adjacent to the U3–18S duplex and mutations in U3 suppress a cold-sensitive mutation in Dhr1. *In vitro*, ATP-dependent strand displacement activity is observed with substrates that mimic U3-pre-rRNA interactions.

### What Is the Nature of the Dhr1 Substrate In Vivo?

RNA helicases generally act on RNP complexes rather than on naked duplex RNA. This is also likely to be the case for Dhr1, as formation of the U3–18S duplex requires additional proteins [11–13] and occurs in the context of the SSU processome, a highly complex assemblage of RNA and protein [35–37]. The genetic suppression of a *dhr1* cold-sensitive mutant by mutations in the region of U3 that base-pairs with the 18S portion of the pre-rRNA provides compelling evidence that the U3–18S duplex is the *in vivo* substrate of Dhr1. However, several of the suppressing mutations affect residues that are not predicted to be involved in base-paired interactions. These include nucleotide G1, at the extreme 5′-end of U3, C11, in the bulge between box A’ and box A in the U3–18S duplex, and A28, adjacent to the duplex predicted to form between box A and nts 1139–1142 of 18S. We suggest that mutations at these positions affect protein-RNA interactions within the pre-ribosome. Partial disruption of these interactions by mutations in U3 may partially overcome defects of a *dhr1* mutant. Proteins that may stabilize U3-pre-rRNA interactions include the U3-associated proteins Imp3 and Imp4. Imp3 is an RNA chaperone that unfolds the 5′-stem loop of U3 to allow its hybridization with 18S *in vitro* [11,13]. The specific function of Imp4 is not known, but it could work in concert with Imp3 to stabilize the short duplex formed between U3 box A and nts 1139–1143 of 18S, on the 3′ side of the CPK (Figs 1 and 6C). We noted that residues C11 and A28 of the U3 contribute to the U3 box A/A’ stem structure that forms when this RNA is not engaged with the pre-rRNA. We considered that Dhr1 might disrupt a competing intramolecular U3 stem-structure, rather than the U3–18S duplex. However, mutation of CGU to UAC at position 36–38 in U3, predicted to destabilize the base of the box A/A’ stem, did not suppress *dhr1-cs* mutant. (S8 Fig.)

### Do DEAH/RHA Box Helicases Use a DEAD Box-Like Mechanism?

We showed that Dhr1 *in vitro* unwinding activity depends on ATP binding but not hydrolysis (Figs 8 and 9). In contrast, rapid product release and enzyme recycling requires ATP hydrolysis. This activity differs from the well-characterized viral DExH helicases that require ATP hydrolysis for processive strand displacement [20,38]. However, it is similar to the behavior of DEAD box proteins that can destabilize and unwind short duplexes prior to ATP hydrolysis [34]. To our knowledge Dhr1 is the first DEAH/RHA helicase for which the mechanistic steps associated with ATP binding and hydrolysis have been identified. We think it is likely that other DEAH/RHA enzymes utilize a similar mechanism with regards to ATP binding and hydrolysis.

Dhr1 cross-linked to U3 at a position immediately 3′ of the U3–18S duplex, identified in our genetic analysis as an *in vivo* target of Dhr1. Because Dhr1 does not appear to be a processive helicase, this raises the question of how Dhr1 acts on the adjacent U3–18S duplex. It should be noted that the CRAC cross-links are not restricted to active site residues within Dhr1. Thus, Dhr1 could be tethered to U3 while disrupting a nearby duplex. Alternatively, Dhr1 may be recruited to the complex by protein interactions that allow it to go through cycles of binding, local duplex unwinding and dissociation, as is the case for DEAD box proteins.

### Dhr1 Identifies an Intermediate of 40S Assembly

Using CRAC experiments, we identified Dhr1 binding sites in 18S rRNA with the highest density of reads in helix 11. Strong cross-linking signals at U319 and U320 indicate direct contact to those positions. Reads also mapped to helices 23 and 44 (Fig. 5G). In the fully folded mature subunit, RNA elements of the platform as well as the top of helix 44 would appear to block access of a bulky helicase to the region of the CPK. In support of this idea, the rRNA region surrounding the CPK was devoid of r-proteins in the Dhr1_K420A_ particle (Fig. 3C, left panel). In particular, the tertiary binding proteins Rps23 (uS12) [27], which lies below the decoding center in the mature subunit, and Rps2 (uS5), which coordinates the CPK, were absent (S1 Table). We therefore envisage that in the U3-bound pre-ribosome, tertiary interactions have not yet been established to allow remodeling of the functional center of the SSU. Notably, intermediates containing an unfolded CPK have also been identified in the bacterial assembly pathway [39]. As Dhr1 acts in the context of the preribosome, perhaps one role of additional SSU processome factors is to hold the rRNA structure in a more open conformation to allow access of U3 snoRNA and enzymes including Dhr1.

Our CRAC data also revealed binding sites that for Dhr1 that are removed from the functional center, the most notable being helix 23 in the platform. This site overlaps the binding site for Rps14. However, Rps14 was present in the Dhr1_K420A_ particle, indicating that its loading was not blocked. Whether or not Dhr1 plays a role at sites other than in the region of the CPK remains to be determined. In addition, while our results demonstrate that Dhr1 is required for U3 unwinding, they do not exclude the possibility that additional factors participate in this process *in vivo*, potentially including another helicase, such as Has1.

### Does Dhr1 Activity Trigger CPK Formation?

The pre-40S particles accumulated in the Dhr1_K420A_ mutant strain retain U3 and the SSU processome, but have largely undergone cleavage at sites A1 and A2. The appearance of high levels of the 21S demonstrates cleavage at site A3 prior to A2 cleavage, strongly indicating delayed A2 processing, and this is supported by the loss of the 27SA2 pre-rRNA in the mutant. We predict that release of U3 normally precedes (and very likely stimulates) pre-rRNA cleavage at site A2. However, in the absence of U3 release some level of pre-rRNA cleavage still occurs. More importantly, it seems clear that the loss of Dhr1 activity not only blocked release of U3, but also of many r-protein factors of the SSU processome. The isolation of U3 suppressors of the Dhr1 mutants suggests that U3 dissociation by Dhr1 is a key step in triggering release of core components of the SSU Processome.

## Materials and Methods

### Plasmids and Strains

Strains and plasmids are listed in S2 and S3 Tables. AJY3324 was derived from the heterozygous diploid deletion collection (Open Biosystems). AJY3335 and AJY3711 were made by integrating the KanMX6- P_GAL1_–3HA cassette from pFA6a-KanMX6-P_GAL1_–3HA [40] into the *DHR1* locus of BY4742 and BY4741, respectively. AJY3583 was made by individually amplifying the P_GAL_-*SNR17A*::*URA3* and *snr17b∆*::*LEU2* loci from YKW100 and integrating them into BY4741. AJY3752 was a haploid spore clone from crossing AJY3335 with AJY3583. pAJ2312 encoded Dhr1 with the C-terminal extension Leu-Glu-6xHis. Mutations in *DHR1* were introduced by site-specific mutagenesis.

### Identification of Cold-Sensitive dhr1 Mutants

*DHR1* was randomly mutagenized by amplification with Taq DNA polymerase using oligonucleotides AJO1566 and AJO1567. The PCR product was co-transformed with MscI digested pAJ2593 into AJY3711. The transformants were selected on synthetic media with galactose as the sole carbon source and lacking uracil. Transformants were screened by replica plating to glucose-containing media lacking uracil at RT. Sequencing *dhr1-cs2* revealed multiple mutations: E330G, K399E, T422A, A557G, T633A, and K892E.

### Identification U3 Mutants

*dhr1-cs2* was introduced into AJY3752 on a Clonat-resistance vector (pAJ3095). The entire insert containing *SNR17A* in pAJ2587 was amplified by PCR using M13 forward and reverse primers with Taq DNA polymerase in buffer containing MnCl_2_ and imbalanced nucleotides to increase the rate of mutagenesis. The PCR product was cotransformed with pAJ2587, digested with SalI and NcoI to remove the entire *SNR17A* gene, into AJY3752 bearing pAJ3095. Transformants were screened for improved growth at 20°C.

### Sucrose Density Gradient Sedimentation

Sucrose density gradient sedimentation was carried out as described previously [41]. Fractions were precipitated with 10% TCA and proteins were separated on 8% SDS-PAGE gels, transferred to a nitrocellulose membrane and subjected to Western blot analysis.

### Immunoprecipitation and Western Blotting

For immunoprecipitations, 250 ml cultures were grown in leu- galactose media to an OD_600_ of 0.08 at 30°C, followed by the addition of 2% glucose and shifted to the appropriate temperature for 6 h. Cells were resuspended in 500 μl of IP buffer (100 mM NaCl, 50 mM Tris-HCl [pH 7.5], 1.5 mM MgCl_2_, 0.15% NP40, 1 mM PMSF, 1 μg/ml leupeptin, 1 μg/ml pepstatin A), lysed by vortexing with glass beads, and clarified by centrifugation at 15,000*g* at 4°C. Immunoprecipitation with the TAP tag was performed by incubating extracts with IgG-Sepharose beads (Amersham IgG Sepharose 6 Fast Flow) for 2 h at 4°C, followed by TEV enzyme cleavage at 16°C for 2 h. The eluted proteins were precipitated by adding 10% TCA, resuspended in Laemmli buffer and separated on an 8% SDS-PAGE gel. Immunoprecipitation for the 13myc tag was performed by incubating extracts with monoclonal (9e10) anti-myc antibody (Covance) for 2 h at 4°C, followed by addition of Protein A-conjugated beads (Milipore) and an additional incubation for 1 h at 4°C. The beads were washed three times and proteins were eluted in Laemmli buffer. Western blotting was done using the indicated antibodies. Cross reaction of anti-Rpl30 antibody was used to detect Rps2.

### Northern Blotting

All RNAs were prepared and Northern blotting was carried out as described previously [41]. Oligonucleotide probes are listed in S4 Table.

### RNA Modification by DMS

Cultures of AJY3711 expressing WT Dhr1 (pAJ2311) or Dhr1_K420A_ (pAJ3081) were grown at 30°C in selective medium containing galactose as the sole carbon source. Glucose was added to repress expression of genomic *DHR1* and after 6.5 h of growth (OD600 ∼0.3), cycloheximide was added to 100 μg/ml final concentration. After an additional 10 min at 30°C cultures were poured over ice and harvested by centrifugation. Extracts were prepared in gradient buffer (20 mM HEPES•KOH [pH 7.6], 50 mM KCL, and 10 mM MgCl_2_) containing 100 μg/ml cycloheximide, 1 mM PMSF, and 1 μM each leupeptin and pepstatin by vortexing with glass beads. Extracts were clarified by centrifugation at 15,000*g* and 25 A_260_ units were loaded onto 7%–47% sucrose gradients prepared in gradient buffer. Samples were centrifuged for 195 min at 40,000*g* in an SW40 rotor. Gradients were fractionated using an ISCO Model 640. Fractions containing 40S were pooled (1.8 ml total). 300 μl aliquots were quickly warmed to RT and 12 μl of ethanol (no DMS control) or DMS diluted in ethanol to 8.3% or 4.2% (v/v) was added (adapted from [42]). After 2 min reactions were quenched by the addition of 15 μl of BME and RNA was precipitated by the addition of 12.5 μg tRNA and 2.5 volumes of ethanol. Pellets were resuspended in LETS (100 mM LiCl, 10 mM EDTA, 10 mM Tris-HCl [pH 7.5], 0.2% SDS) and extracted twice with phenol/CHCl_3_, once with CHCl_3_, and RNA was precipitated with 10% volume of 5 M LiCl and three volumes of ethanol. Pellets were washed with 80% ethanol and RNA was resuspended in water. Reverse transcription was carried out as described [43] using oligonucleotide AJO1849. pAJ2158 was used as a template for a DNA sequencing ladder. Samples were analyzed on an 8% denaturing polyacrylamide gel and imaged by phosphoimaging on a Typhoon FLA 9500. Quantification was done using NIH ImageJ.

### CRAC Experiments

CRAC was performed as previously described [31]. The sequencing data were processed using pyCRAC [44]. Briefly, adapter sequences were removed using flexbar [45] and reads were collapsed (pyFastqDuplicateRemover) to remove potential PCR duplicates. The resulting sequences were aligned to the yeast genome (ENSEMBL, version EF.59) using novoalign 2.07 (www.novocraft.com). Histograms were generated using pyReadCounters and pyPileup.

### Protein Purification

Imp3 was purified as described [13]. WT and mutant Dhr1 proteins were expressed from pAJ2312, pAJ2396, or pAJ3257 overnight at 15°C in BL21 Star (DE3) (Life Technologies) cells supplemented with a vector, which coded for the rare tRNA^Arg^, tRNA^Ile^, and tRNA^Leu^. Cells were washed once and resuspended with extraction buffer (50 mM Tris-HCl [pH 8.0], 500 mM NaCl, 10% [v/v] glycerol, 5 mM BME, 7 units/ml RNase A and 10 units/ml RNase I). The extensive RNase treatment ensured the removal of tightly bound RNA. French press was used to lyse cells and cell extracts were clarified for 10 min at 10,000*g* followed by 30 min at 50,000*g*. Supernatant was loaded on a Ni-NTA resin (Invitrogen) and washed once with extraction buffer without RNase. The resin was then resuspended with 3 column volumes (CV) of extraction buffer and incubated 15 min. The resin was washed extensively with extraction buffer without RNase and protein was eluted with extraction buffer in which NaCl was replaced with 250 mM imidazole. Fractions containing Dhr1 were pooled, supplemented with 1 mM DTT, and applied to SP Hitrap column (GE Healthcare Life Sciences). The column was washed with Buffer A (30 mM Tris [pH 8.0], 5% [v/v] glycerol, 5 mM sodium acetate, and 1 mM DTT). Protein was eluted with a 21 CV gradient from 0% to 60% buffer B (buffer A plus 1 M NaCl). Dhr1 containing fractions were pooled, dialyzed (30 mM Tris [pH 8.0], 10% [v/v] glycerol, 5 mM sodium acetate, 150 mM NaCl, and 1 mM DTT), and concentrated to ∼5 μM. Aliquots were flash frozen and stored at −80°C (S6 Fig.). Yield for WT and mutant Dhr1 was approximately 0.5 mg/liter.

### Mass-Spectrometric Analysis of Dhr1_K420A_ Particles

The yeast strain AJY3711 containing pAJ3090 (dhr1_K420A_-TEV-13myc LEU2 CEN) or pAJ3100 (dhr1_K420A_ LEU2 CEN) was grown in SD Leu- containing 2% glucose for 6 h to deplete endogenouse Dhr1. Extracts were prepared and immunoprecipitation was carried out as described under “Immunoprecipitation and Western Blotting” except Protein-G magnetic Beads (Pierce) were used. After binding for 2 h, beads were washed three times, resuspended in 100 μl of the extraction buffer, and TEV was added. Samples were incubated for 2 h at 16°C with gentle rotation. The supernatants were layered onto 100 μl sucrose cushions (50 mM Tris-HCl [pH 7.5], 100 mM NaCl, 1.5 mM MgCl_2_, 15% sucrose) and centrifuged in a TLA100 rotor (Beckman) for 15 min at 70,000 rpm at 4°C. The pellet was resuspended in 100 mM Tris-HCl (pH8), 4% SDS. Samples were run into a 7% Mini-Protean TGX polyacrylamide gel (BioRad) for 5 min 100 V and stained with Imperial Protein stain (Thermo Scientific). The protein-containing band was diced and prepared for in-gel digestion essentially as in [46]. After standard acetonitrile elution of digested peptides, the gel pieces were swelled in 6 M urea and eluted with 75% acetonitrile. The combined eluate volume was reduced by SpeedVac centrifugation and digested peptides purified on HyperSep C-18 SpinTips (Thermo Scientific).

### Proteomics Data Acquisition and Data Analysis

Peptides were separated on a reverse-phase Zorbax C18 column (Agilent) using a 5%–38% acetonitrile gradient over 137 min and subjected to nanoelectrospray-ionization tandem mass spectrometry on an LTQ-Orbitrap (Thermo Scientific) with parameters as in [47]. Resulting spectra were searched against the UniProtKB YEAST *Saccharomyces cerevisiae* (strain ATCC 204508 / S288c) FASTA using Sequest HT in the Proteome Discoverer v1.4 software (Thermo Scientific). High confidence peptide spectrum matches were filtered at <1% FDR using Percolator. Significance parameters were as in [47].

### In Vitro Assays

**RNA substrates**. All RNAs were as previously described [11–13] and were purified by gel electrophoresis and refolded except for the ETS2 and 18S oligomers.

**ATPase assay**. All the reactions were performed at RT. Dhr1 was pre-incubated in reaction buffer (20 mM Tris [pH 8.0], 40 mM KCl, 2 mM DTT), with either U3 snoRNA or poly(A) for 2 min and in some reactions Imp3 was added (0.5 μM final concentration). Reactions were initiated by rapid addition and mixing of equimolar mixture of ATP and MgCl_2_ with trace [γ-^32^P]ATP. Final reaction conditions were 1 mM ATP, 1 mM MgCl_2_, 0.5 μM Dhr1, 60 mM Tris (pH 8.0), 40 mM KCl, 15 mM NaCl, 4% (v/v) glycerol, 2 mM sodium acetate and 2.4 mM DTT. For reaction with RNA, final concentration was either 60 μM U3 snoRNA or 4 mg/ml poly(A). Aliquots of the reaction mixtures were withdrawn at different time intervals and quenched by addition of 3 volumes of stop buffer (90% [v/v] formamide, 50 mM EDTA). To separate reaction products 0.8 μl of quenched sample was spotted on thin layer chromatography (TLC) plates, dried, and developed for 10 min with developing buffer (0.8 M acetic acid, 0.8 M LiCl). The TLC plate was then exposed on a Fuji imaging plate (BAS 2024), scanned by a Typhoon 9400 (Amersham Biosciences, GE), and quantified using Image Quant TL 7.0 (GE Healthcase Life Sciences). Normalized band intensity quantification was displayed using PRISM 6 (GraphPad, Inc.). Kinetic parameters of Dhr1 were determined by fitting the initial ATPase hydrolysis activity dependence on ATP concentration to the Michaelis–Menten equation using PRISM 6.

**Unwinding reactions**. All unwinding reactions were performed at RT. Pre-steady state U3–18S reactions: to pre-form U3–18S duplex, U3 MINI was incubated with Imp3 for 10 min [12]. After addition of ^32^P-18S the reaction was further incubated for 30 min. The pre-formed U3–18S duplex was then incubated with Dhr1 for 5 min. Reactions were initiated by rapid addition and mixing of equimolar mixture of ATP and MgCl_2_. Final concentrations were 1 mM ATP, 1.5 mM MgCl_2_, 40 nM U3 MINI, ≤1.2 nM ^32^P 18S, 1.2 μM Imp3, 0.5 μM Dhr1, 60 mM Tris (pH 8.0), 40 mM KCl, 15 mM NaCl, 4% (v/v) glycerol, 2 mM sodium acetate, 50 mM urea, 2.4 mM DTT, 0.2 mg/ml BSA and 0.8 units/μl RNasin. Aliquots were withdrawn at different times and quenched on ice. Ice was used instead of a stop solution containing SDS because this detergent removes Imp3, resulting in the release of 18S RNA.

Pre-steady state U3-ETS2 reactions: to form the U3-ETS2 duplex, U3 was first incubated with ^32^P-ETS2 for 10 min followed by 10 min with buffer supplemented with MgCl_2_. The pre-formed U3-ETS2 duplex was incubated with Dhr1 for 5 min and reactions were initiated by rapid addition of 1 mM mixture of ATP and MgCl_2_. The final concentrations were 1 mM ATP, 1.5 mM MgCl_2_, 1 nM U3 snoRNA, ≤0.3 nM ^32^P ETS2, 0.5 μM Dhr1, 25 mM Tris (pH 8.0), 40 mM KCl, 15 mM NaCl, 7% (v/v) glycerol, 2.4 mM DTT, 0.8 units/μl RNasin, and 0.2 mg/ml BSA. Reactions were sampled and quenched by addition of one-half volume of stop buffer (150 mM Tris [pH 8.0], 0.3% [w/v] SDS, and 150 mM EDTA).

All unwinding reactions in the strand exchange regime were performed at RT. Steady state U3-ETS2 reactions: the reaction procedure was essentially the same as for pre-steady state reactions described above, but with several differences. Instead of using U3 snoRNA, 5 μM U3 (1–76) was incubated with 0.5 μM ETS2 with trace ^32^P-ETS2 to form the U3-ETS2 duplex. Activity under these conditions was not observed with full length U3 snoRNA, presumably owing to non-specific binding of Dhr1 to the 3′ region of U3 that includes the K-turn motifs in box B/C and box C/D. After pre-incubating the duplex with 0.1 μM Dhr1, reactions were initiated by rapid addition of 5 μM ETS2 and 6 mM mixture of ATP and MgCl_2_. The remaining buffer conditions are the same as those in the pre-steady reactions. The amount of duplex and single stranded RNA for both of the above helicase reactions were resolved by EMSAs [12].

## Supporting Information

S1 Data Supplemental data for Fig 2A and 2B. UV traces of sucrose gradients.
(XLSX)

S2 Data Supplemental data for Fig. 5. CRAC reads.
(XLSX)

S3 Data Supplemental data for Fig. 7A. Dhr1 ATPase activity.
(XLSX)

S4 Data Supplemental data for Figs 7C and S7. Kinetic parameters.
(XLSX)

S5 Data Supplemental data for Fig. 8D. U3-ETS2 Unwinding assay [E] > [duplex].
(XLSX)

S6 Data Supplemental data for Fig. 8f. U3-ETS2 Unwinding assay [duplex] > [E].
(XLSX)

S7 Data Supplemental data for Fig. 9D. U3–18S Unwinding assay.
(XLSX)

S1 Fig Time course of Dhr1 depletion in glucose. (A) 10-fold serial dilutions of yeast strains BY4741 (WT) and AJY3711 (P_GAL1_-HA-*DHR1*) were spotted onto YP-galactose (galactose) and YPD (glucose) and incubated at 30°C for 2 days. (B) Strain AJY3711 was cultured in YP-galactose. At time zero, glucose was added to 2% final concentration. Samples were taken at the indicated times, proteins extracts were made from a constant number of cells and SDS-PAGE and Western blotting as done to detect HA-tagged Dhr1.
(TIF)

S2 Fig Growth assays of Dhr1_K420A_-13myc and Dhr1–6His. (A) 10-fold serial dilutions of yeast strain AJY3711 containing plasmid pAJ2311 (*DHR1*–13myc) or pAJ3081 (*dhr1*_*K420A*_-13myc) were spotted onto SD Leu- galactose (galactose) and SD Leu- glucose (glucose) and incubated at 30°C for 2 days. (B) 10-fold serial dilutions of yeast strain AJY3711 containing plasmids pRS315 (vector), pAJ3082 (*DHR1* untagged), or pAJ3317 (*DHR1*–6His) were spotted onto SD Leu- glucose and incubated at 30°C for 2 days.
(TIF)

S3 Fig Dhr1-K420A co-immunoprecipitates U3-associated proteins. (A) Cultures of AJY3711 (P_GAL1_–3xHA-*DHR1*) expressing untagged WT *DHR1* (pAJ3082), WT *DHR1*–13myc (pAJ2311), or *dhr1*_*K420A*_-13myc (pAJ3081) were shifted to glucose media for 6 h to deplete 3xHA-Dhr1. Proteins were immunoprecipitated from whole cell extracts with anti-myc antibody and subjected to SDS-PAGE and Western blotting as described in the legend of Fig. 2.
(TIF)

S4 Fig Dhr1 preferentially cross-links to helices (H) 11, 23, and 44 in the 18S rRNA. (A) The reads from Dhr1-CRAC mapped to the rRNA are plotted. Read coverage indicates the total number of reads that cover each nucleotide in the rRNA. A schematic representation of an rDNA repeat is indicated below the plot. Helices H11, H23, and H44 that were recovered from both experiments are indicated. Peaks that are frequently recovered in control samples are indicated with an asterisk. (B) Read densities of efficiently cross-linked regions (helices 11, 23, and 44) in the crystal structure of the yeast 18S rRNA [28]. The colors indicate the read density covering the rRNA region. Relevant rRNA helices and read densities are indicated. See S5 Fig. for a complete overview of read distributions for each nucleotide in the 18S rRNA secondary structure. (C) The reads from the control experiment that mapped to the rRNA are plotted as described in (A). Helices that are reproducibly recovered from CRAC control experiments [32,48] are indicated with “H.” Additional supporting data are provided in S2 Data.
(TIF)

S5 Fig Read distributions for each nucleotide in the 18S rRNA secondary structure. Shown is a secondary structure model for the *S*. *cerevisiae* 18S rRNA (available at http://www.rna.ccbb.utexas.edu). Colors indicate the average percentage of read coverage (*n* = 2) for each nucleotide in the 18S rRNA. Helices where consistently high cross-linking signals were observed are indicated with “H.” Additional supporting data are provided in S2 Data.
(TIF)

S6 Fig U3 sedimentation in *dhr1-cs2*. (A) 10-fold serial dilutions of AJY3715 (*dhr1∆*::*KanMX*) containing pAJ2593 (*DHR1-WT*) or pAJ2388 (*dhr1-cs2*) were spotted onto Ura-medium and incubated for 4 days at 20°C or 30°C. (B) Strain AJY3711 carrying plasmid pAJ2388 was cultured in SD Ura- galactose at 30°C. Glucose was added to 2% final concentration and cells were cultured for an additional 6 h at 20°C. Extracts were prepared and fractionated through 7%–47% sucrose gradients, RNA isolated, and Northern blotting for U3 were as described for Fig. 2.
(TIF)

S7 Fig Steady state kinetic data of ATPase activity catalyzed by Dhr1 and mutants. Initial velocities of P_i_ released after addition of ATP at RT in the presence of poly(A). ATP hydrolysis activity is plotted as a function of ATP concentration with either Dhr1 (purple), Dhr1_D516A/E517A_ (blue), or Dhr1_K420A_ (green). Additional supporting data are provided in S3 and S4 Data.
(TIF)

S8 Fig Mutation of U3 nt 36–38 (CGU to UAC) does not suppress the *dhr1-cs2* mutant. Nucleotides CGU at position 36 to 38 of U3 were mutated to UAC in pAJ2587. WT and mutant U3 were expressed in AJY3752 (*P*_*GAL*_-*DHR1 P*_*GAL*_-*SNR17A snr17B∆*) containing pAJ3095 (*dhr1-cs2*) and the ability of mutant U3 to suppress the cold-sensitive growth of *dhr1-cs2* was assayed by serial dilution on His- glucose medium. Plates were incubated at 20°C for 6 days.
(TIF)

S1 Table Mass spectrometric analysis of Dhr1_K420A_ particle. Mass spectrometric analysis was carried out on affinity purified Dhr1K420A (TEV-13xmyc-tagged) and mock (untagged) particles. Affinity purified particles were subjected to in-gel trypsin digestion and peptides identified by mass spectrometry. The number of peptide spectral matches (Σ #PSM) and molecular weight (MW) are given. Hits are grouped into known complexes and color-coded. As a rough proxy for abundance, PSM was divided by molecular mass and normalized to the value for Dhr1 (PSM/MW).
(DOCX)

S2 Table Strains used in this work. (DOCX)

S3 Table Plasmids used in this work. (DOCX)

S4 Table Oligonucleotides used in this work. (DOCX)

## Abbreviations

CPK: central pseudoknot
CRAC: cross-linking and analysis of cDNA
DMS: dimethyl sulfate
EMSA: electrophoretic mobility shift assay
ETS: external-transcribed spacer
MS: mass spectrometry
pre-rRNA: pre-ribosomal RNA
r-protein: ribosomal protein
RT: room temperature
snoRNA: small nucleolar RNA
SSU: small ribosomal subunit
TEV: tobacco etch virus protease
WT: wild type

## References

[1] McCutcheon JP, Moran NA (2012) Extreme genome reduction in symbiotic bacteria. Nat Rev Microbiol 10: 13–26. 10.1038/nrmicro2670 22064560

[2] Traub P, Nomura M (1968) Structure and function of E. coli ribosomes. V. Reconstitution of functionally active 30S ribosomal particles from RNA and proteins. Proc Natl Acad Sci U S A 59: 777–784. 4868216 10.1073/pnas.59.3.777 PMC224743

[3] Dohme F, Nierhaus KH (1976) Role of 5S RNA in assembly and function of the 50S subunit from Escherichia coli. Proc Natl Acad Sci U S A 73: 2221–2225. 781671 10.1073/pnas.73.7.2221 PMC430504

[4] Fromont-Racine M, Senger B, Saveanu C, Fasiolo F (2003) Ribosome assembly in eukaryotes. Gene 313: 17–42. 12957375 10.1016/s0378-1119(03)00629-2

[5] Woolford JL Jr., Baserga SJ (2013) Ribosome biogenesis in the yeast Saccharomyces cerevisiae. Genetics 195: 643–681. 10.1534/genetics.113.153197 24190922 PMC3813855

[6] Borovjagin AV, Gerbi SA (1999) U3 small nucleolar RNA is essential for cleavage at sites 1, 2 and 3 in pre-rRNA and determines which rRNA processing pathway is taken in Xenopus oocytes. J Mol Biol 286: 1347–1363. 10064702 10.1006/jmbi.1999.2527

[7] Dutca LM, Gallagher JE, Baserga SJ (2011) The initial U3 snoRNA:pre-rRNA base pairing interaction required for pre-18S rRNA folding revealed by in vivo chemical probing. Nucleic Acids Res 39: 5164–5180. 10.1093/nar/gkr044 21349877 PMC3130255

[8] Hughes JM (1996) Functional base-pairing interaction between highly conserved elements of U3 small nucleolar RNA and the small ribosomal subunit RNA. J Mol Biol 259: 645–654. 8683571 10.1006/jmbi.1996.0346

[9] Marmier-Gourrier N, Clery A, Schlotter F, Senty-Segault V, Branlant C (2011) A second base pair interaction between U3 small nucleolar RNA and the 5′-ETS region is required for early cleavage of the yeast pre-ribosomal RNA. Nucleic Acids Res 39: 9731–9745. 10.1093/nar/gkr675 21890904 PMC3239212

[10] Sharma K, Tollervey D (1999) Base pairing between U3 small nucleolar RNA and the 5′ end of 18S rRNA is required for pre-rRNA processing. Mol Cell Biol 19: 6012–6019. 10454548 10.1128/mcb.19.9.6012 PMC84488

[11] Gerczei T, Correll CC (2004) Imp3p and Imp4p mediate formation of essential U3-precursor rRNA (pre-rRNA) duplexes, possibly to recruit the small subunit processome to the pre-rRNA. Proc Natl Acad Sci U S A 101: 15301–15306. 15489263 10.1073/pnas.0406819101 PMC524450

[12] Gerczei T, Shah BN, Manzo AJ, Walter NG, Correll CC (2009) RNA chaperones stimulate formation and yield of the U3 snoRNA-Pre-rRNA duplexes needed for eukaryotic ribosome biogenesis. J Mol Biol 390: 991–1006. 10.1016/j.jmb.2009.05.072 19482034 PMC2881153

[13] Shah BN, Liu X, Correll CC (2013) Imp3 unfolds stem structures in pre-rRNA and U3 snoRNA to form a duplex essential for small subunit processing. RNA 19: 1372–1383. 10.1261/rna.039511.113 23980203 PMC3854528

[14] Venema J, Tollervey D (1999) Ribosome synthesis in Saccharomyces cerevisiae. Annu Rev Genet 33: 261–311. 10690410 10.1146/annurev.genet.33.1.261

[15] Henras AK, Soudet J, Gerus M, Lebaron S, Caizergues-Ferrer M, et al (2008) The post-transcriptional steps of eukaryotic ribosome biogenesis. Cell Mol Life Sci 65: 2334–2359. 10.1007/s00018-008-8027-0 18408888 PMC11131730

[16] Kos M, Tollervey D (2010) Yeast pre-rRNA processing and modification occur cotranscriptionally. Mol Cell 37: 809–820. 10.1016/j.molcel.2010.02.024 20347423 PMC2860240

[17] Bernstein KA, Granneman S, Lee AV, Manickam S, Baserga SJ (2006) Comprehensive mutational analysis of yeast DEXD/H box RNA helicases involved in large ribosomal subunit biogenesis. Mol Cell Biol 26: 1195–1208. 16449635 10.1128/MCB.26.4.1195-1208.2006 PMC1367183

[18] Granneman S, Bernstein KA, Bleichert F, Baserga SJ (2006) Comprehensive mutational analysis of yeast DEXD/H box RNA helicases required for small ribosomal subunit synthesis. Mol Cell Biol 26: 1183–1194. 16449634 10.1128/MCB.26.4.1183-1194.2006 PMC1367182

[19] Rodriguez-Galan O, Garcia-Gomez JJ, de la Cruz J (2013) Yeast and human RNA helicases involved in ribosome biogenesis: current status and perspectives. Biochim Biophys Acta 1829: 775–790. 10.1016/j.bbagrm.2013.01.007 23357782

[20] Jankowsky E (2011) RNA helicases at work: binding and rearranging. Trends Biochem Sci 36: 19–29. 10.1016/j.tibs.2010.07.008 20813532 PMC3017212

[21] Pyle AM (2008) Translocation and unwinding mechanisms of RNA and DNA helicases. Annu Rev Biophys 37: 317–336. 10.1146/annurev.biophys.37.032807.125908 18573084

[22] Colley A, Beggs JD, Tollervey D, Lafontaine DL (2000) Dhr1p, a putative DEAH-box RNA helicase, is associated with the box C+D snoRNP U3. Mol Cell Biol 20: 7238–7246. 10982841 10.1128/mcb.20.19.7238-7246.2000 PMC86278

[23] Liang XH, Fournier MJ (2006) The helicase Has1p is required for snoRNA release from pre-rRNA. Mol Cell Biol 26: 7437–7450. 16908538 10.1128/MCB.00664-06 PMC1636851

[24] Horn DM, Mason SL, Karbstein K (2011) Rcl1 protein, a novel nuclease for 18 S ribosomal RNA production. J Biol Chem 286: 34082–34087. 10.1074/jbc.M111.268649 21849504 PMC3190816

[25] Januszyk K, Lima CD (2014) The eukaryotic RNA exosome. Curr Opin Struct Biol 24C: 132–140. 10.1016/j.sbi.2014.01.011 PMC3985421 24525139

[26] Perez-Fernandez J, Martin-Marcos P, Dosil M (2011) Elucidation of the assembly events required for the recruitment of Utp20, Imp4 and Bms1 onto nascent pre-ribosomes. Nucleic Acids Res 39: 8105–8121. 10.1093/nar/gkr508 21724601 PMC3185420

[27] Shajani Z, Sykes MT, Williamson JR (2011) Assembly of bacterial ribosomes. Annu Rev Biochem 80: 501–526. 10.1146/annurev-biochem-062608-160432 21529161

[28] Ben-Shem A, Garreau de Loubresse N, Melnikov S, Jenner L, Yusupova G, et al (2011) The structure of the eukaryotic ribosome at 3.0 A resolution. Science 334: 1524–1529. 10.1126/science.1212642 22096102

[29] Swiatkowska A, Wlotzka W, Tuck A, Barrass JD, Beggs JD, et al (2012) Kinetic analysis of pre-ribosome structure in vivo. RNA 18: 2187–2200. 10.1261/rna.034751.112 23093724 PMC3504671

[30] Gregory ST, Dahlberg AE (2009) Genetic and structural analysis of base substitutions in the central pseudoknot of Thermus thermophilus 16S ribosomal RNA. RNA 15: 215–223. 10.1261/rna.1374809 19144908 PMC2648708

[31] Granneman S, Kudla G, Petfalski E, Tollervey D (2009) Identification of protein binding sites on U3 snoRNA and pre-rRNA by UV cross-linking and high-throughput analysis of cDNAs. Proc Natl Acad Sci U S A 106: 9613–9618. 10.1073/pnas.0901997106 19482942 PMC2688437

[32] Granneman S, Petfalski E, Tollervey D (2011) A cluster of ribosome synthesis factors regulate pre-rRNA folding and 5.8S rRNA maturation by the Rat1 exonuclease. EMBO J 30: 4006–4019. 10.1038/emboj.2011.256 21811236 PMC3209772

[33] Jankowsky E, Putnam A (2010) Duplex unwinding with DEAD-box proteins. Methods Mol Biol 587: 245–264. 10.1007/978-1-60327-355-8_18 20225155

[34] Liu F, Putnam A, Jankowsky E (2008) ATP hydrolysis is required for DEAD-box protein recycling but not for duplex unwinding. Proc Natl Acad Sci U S A 105: 20209–20214. 10.1073/pnas.0811115106 19088201 PMC2629341

[35] Dragon F, Gallagher JE, Compagnone-Post PA, Mitchell BM, Porwancher KA, et al (2002) A large nucleolar U3 ribonucleoprotein required for 18S ribosomal RNA biogenesis. Nature 417: 967–970. 12068309 10.1038/nature00769 PMC11487672

[36] Grandi P, Rybin V, Bassler J, Petfalski E, Strauss D, et al (2002) 90S pre-ribosomes include the 35S pre-rRNA, the U3 snoRNP, and 40S subunit processing factors but predominantly lack 60S synthesis factors. Mol Cell 10: 105–115. 12150911 10.1016/s1097-2765(02)00579-8

[37] Schafer T, Strauss D, Petfalski E, Tollervey D, Hurt E (2003) The path from nucleolar 90S to cytoplasmic 40S pre-ribosomes. EMBO J 22: 1370–1380. 12628929 10.1093/emboj/cdg121 PMC151049

[38] Jankowsky E, Gross CH, Shuman S, Pyle AM (2000) The DExH protein NPH-II is a processive and directional motor for unwinding RNA. Nature 403: 447–451. 10667799 10.1038/35000239

[39] Clatterbuck Soper SF, Dator RP, Limbach PA, Woodson SA (2013) In vivo X-ray footprinting of pre-30S ribosomes reveals chaperone-dependent remodeling of late assembly intermediates. Mol Cell 52: 506–516. 10.1016/j.molcel.2013.09.020 24207057 PMC3840108

[40] Longtine MS, McKenzie A 3rd, Demarini DJ, Shah NG, Wach A, et al (1998) Additional modules for versatile and economical PCR-based gene deletion and modification in Saccharomyces cerevisiae. Yeast 14: 953–961. 9717241 10.1002/(SICI)1097-0061(199807)14:10<953::AID-YEA293>3.0.CO;2-U

[41] Sardana R, White JP, Johnson AW (2013) The rRNA methyltransferase Bud23 shows functional interaction with components of the SSU processome and RNase MRP. RNA 19: 828–840. 10.1261/rna.037671.112 23604635 PMC3683916

[42] Tijerina P, Mohr S, Russell R (2007) DMS footprinting of structured RNAs and RNA-protein complexes. Nat Protoc 2: 2608–2623. 17948004 10.1038/nprot.2007.380 PMC2701642

[43] Sengupta J, Bussiere C, Pallesen J, West M, Johnson AW, et al (2010) Characterization of the nuclear export adaptor protein Nmd3 in association with the 60S ribosomal subunit. J Cell Biol 189: 1079–1086. 10.1083/jcb.201001124 20584915 PMC2894450

[44] Webb S, Hector RD, Kudla G, Granneman S (2014) PAR-CLIP data indicate that Nrd1-Nab3-dependent transcription termination regulates expression of hundreds of protein coding genes in yeast. Genome Biol 15: R8 10.1186/gb-2014-15-1-r8 24393166 PMC4053934

[45] Dodt M, Roehr JT, Ahmed R, Dieterich C (2012) Flexbar − flexible barcode and adapter processing for next-generation sequencing platforms. MDPIBiology 1: 895–905. 10.3390/biology1030895 PMC4009805 24832523

[46] Shevchenko A, Tomas H, Havlis J, Olsen JV, Mann M (2006) In-gel digestion for mass spectrometric characterization of proteins and proteomes. Nat Protoc 1: 2856–2860. 17406544 10.1038/nprot.2006.468

[47] Boutz DR, Collins PJ, Suresh U, Lu M, Ramirez CM, et al (2011) Two-tiered approach identifies a network of cancer and liver disease-related genes regulated by miR-122. J Biol Chem 286: 18066–18078. 10.1074/jbc.M110.196451 21402708 PMC3093880

[48] Granneman S, Petfalski E, Swiatkowska A, Tollervey D (2010) Cracking pre-40S ribosomal subunit structure by systematic analyses of RNA-protein cross-linking. EMBO J 29: 2026–2036. 10.1038/emboj.2010.86 20453830 PMC2892368

