# Supplementary figures and images for: The DEAH-box Helicase Dhr1 Dissociates U3 from the Pre-rRNA to Promote Formation of the Central Pseudoknot
Source: PLoS Biol. 2015 Feb 24;13(2):e1002083. doi: 10.1371/journal.pbio.1002083 (PMC4340053; doi:10.1371/journal.pbio.1002083)

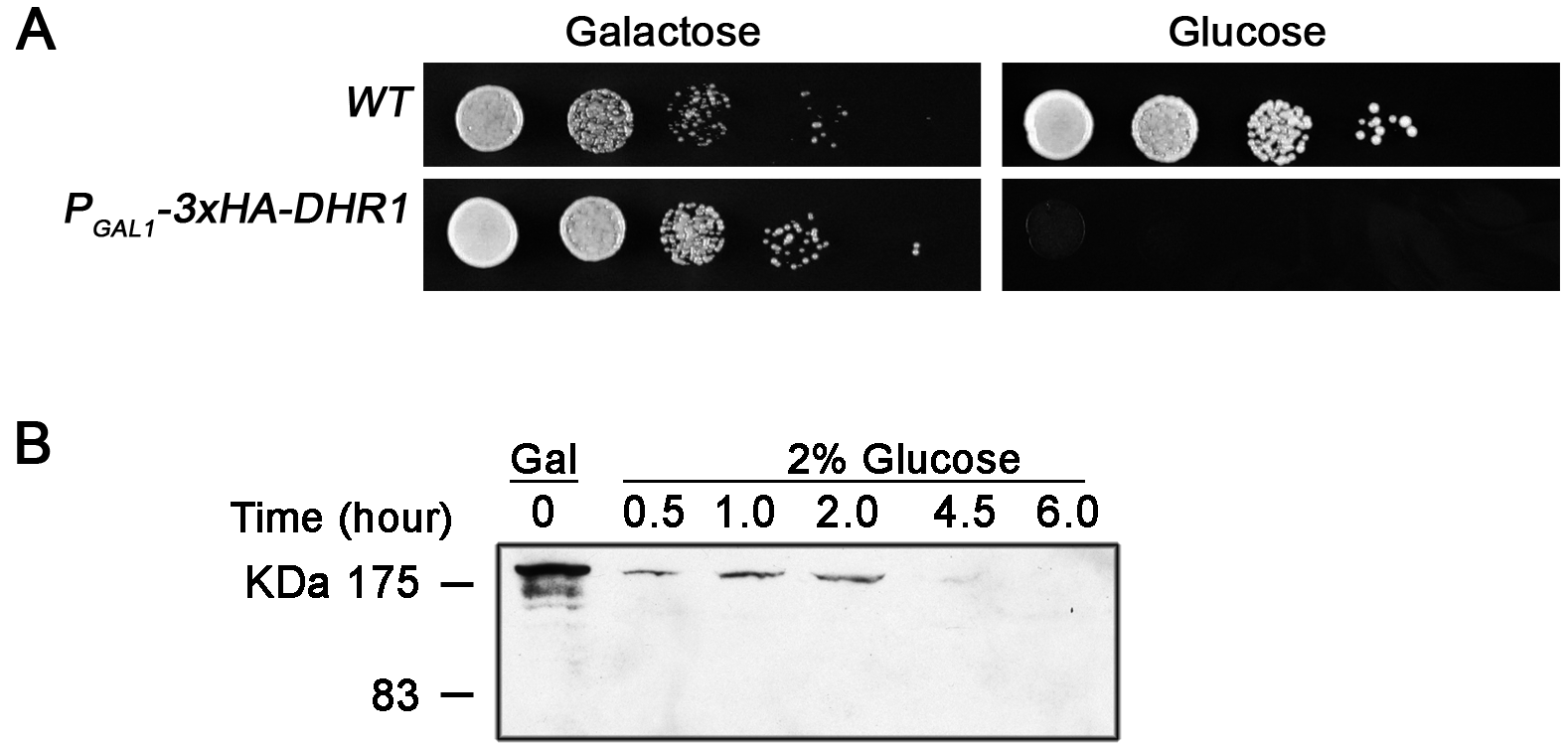

Supplement: S1 Fig — (A) 10-fold serial dilutions of yeast strains BY4741 (WT) and AJY3711 (PGAL1-HA-DHR1) were spotted onto YP-galactose (galactose) and YPD (glucose) and incubated at 30°C for 2 days. (B) Strain AJY3711 was cultured in YP-galactose. At time zero, glucose was added to 2% final concentration. Samples were taken at the indicated times, proteins extracts were made from a constant number of cells and SDS-PAGE and Western blotting as done to detect HA-tagged Dhr1. (TIF) [file pbio.1002083.s008.tif]

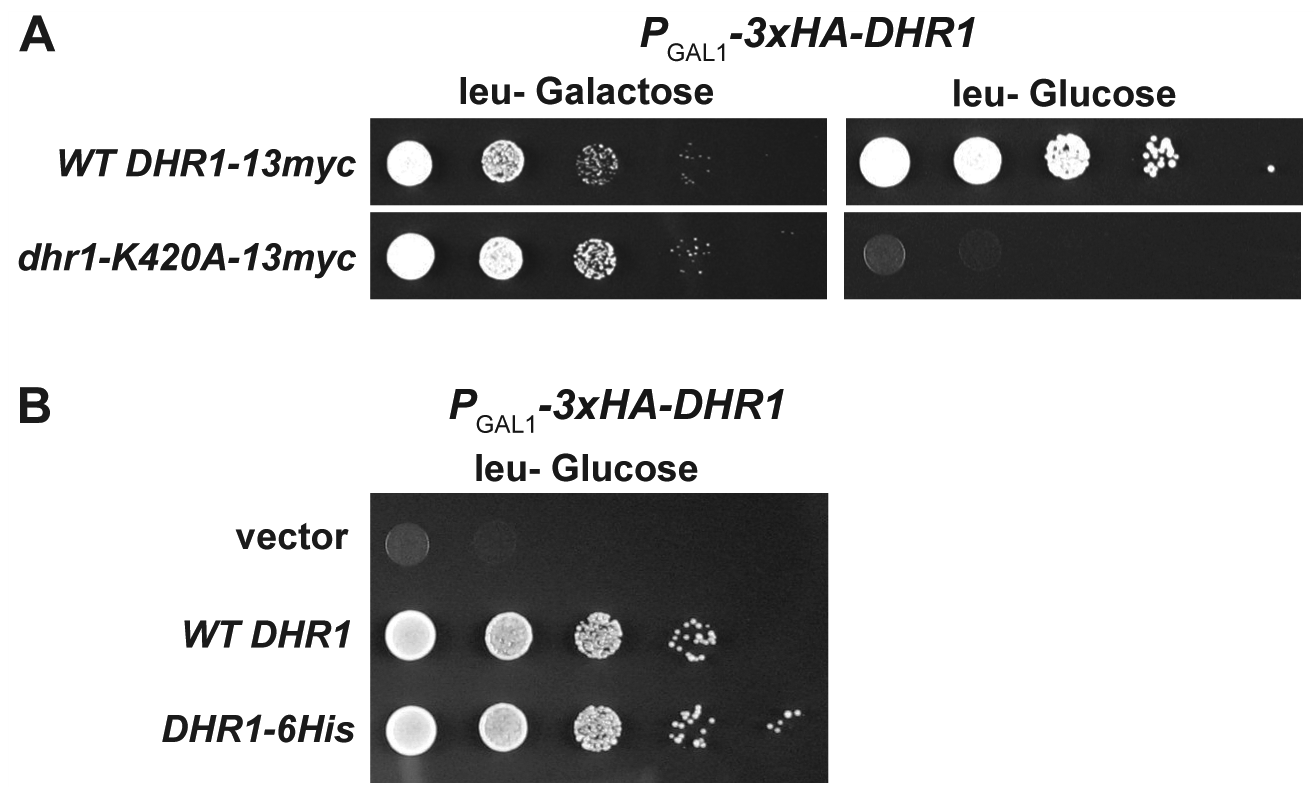

Supplement: S2 Fig — (A) 10-fold serial dilutions of yeast strain AJY3711 containing plasmid pAJ2311 (DHR1–13myc) or pAJ3081 (dhr1 K420A-13myc) were spotted onto SD Leu- galactose (galactose) and SD Leu- glucose (glucose) and incubated at 30°C for 2 days. (B) 10-fold serial dilutions of yeast strain AJY3711 containing plasmids pRS315 (vector), pAJ3082 (DHR1 untagged), or pAJ3317 (DHR1–6His) were spotted onto SD Leu- glucose and incubated at 30°C for 2 days. (TIF) [file pbio.1002083.s009.tif]

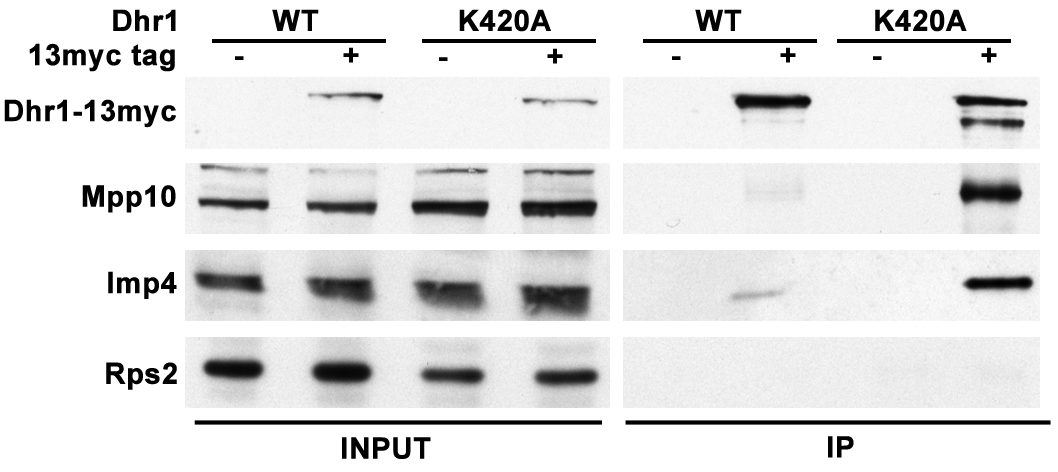

Supplement: S3 Fig — (A) Cultures of AJY3711 (PGAL1–3xHA-DHR1) expressing untagged WT DHR1 (pAJ3082), WT DHR1–13myc (pAJ2311), or dhr1 K420A-13myc (pAJ3081) were shifted to glucose media for 6 h to deplete 3xHA-Dhr1. Proteins were immunoprecipitated from whole cell extracts with anti-myc antibody and subjected to SDS-PAGE and Western blotting as described in the legend of Fig. 2. (TIF) [file pbio.1002083.s010.tif]

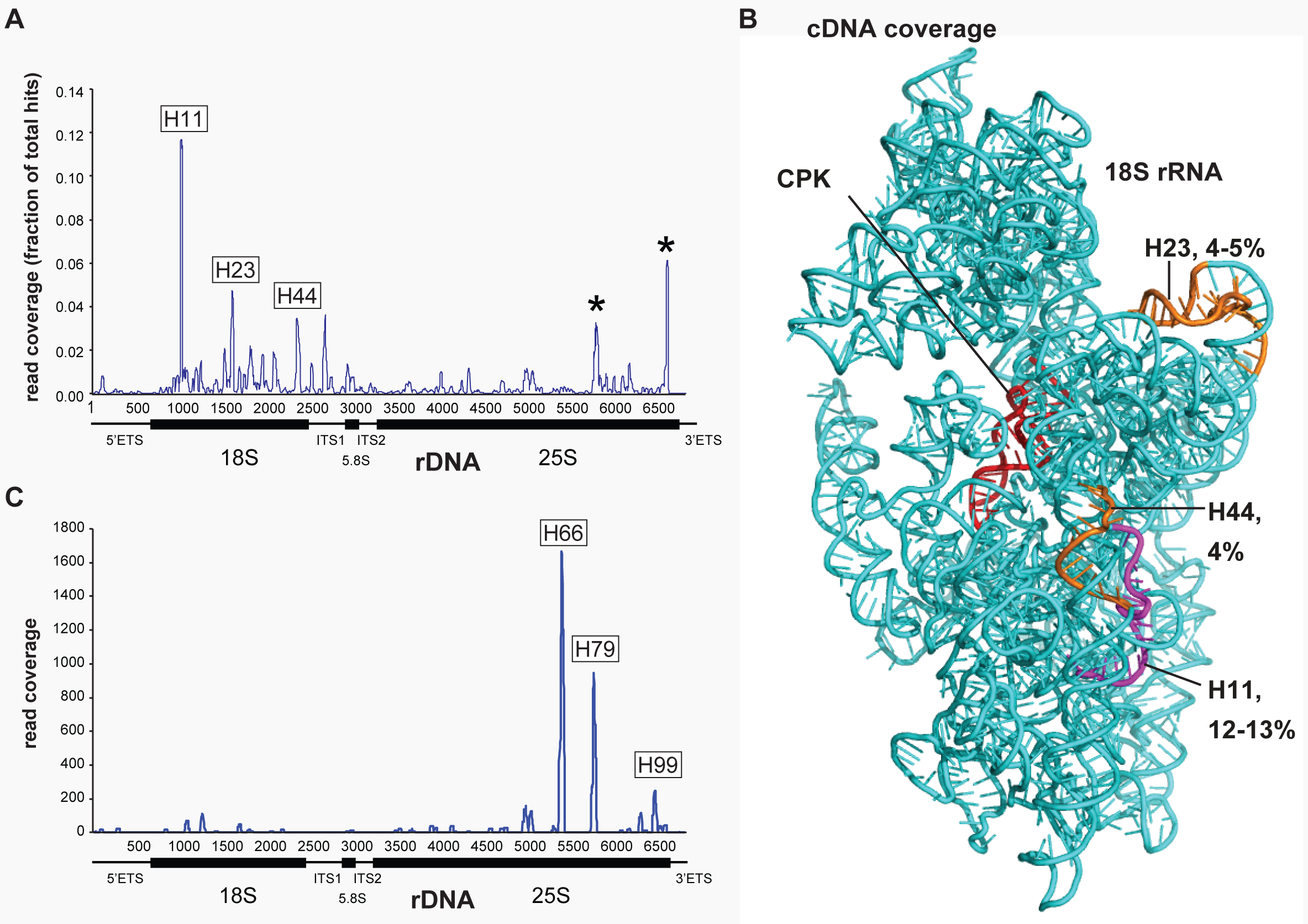

Supplement: S4 Fig — (A) The reads from Dhr1-CRAC mapped to the rRNA are plotted. Read coverage indicates the total number of reads that cover each nucleotide in the rRNA. A schematic representation of an rDNA repeat is indicated below the plot. Helices H11, H23, and H44 that were recovered from both experiments are indicated. Peaks that are frequently recovered in control samples are indicated with an asterisk. (B) Read densities of efficiently cross-linked regions (helices 11, 23, and 44) in the crystal structure of the yeast 18S rRNA [28]. The colors indicate the read density covering the rRNA region. Relevant rRNA helices and read densities are indicated. See S5 Fig. for a complete overview of read distributions for each nucleotide in the 18S rRNA secondary structure. (C) The reads from the control experiment that mapped to the rRNA are plotted as described in (A). Helices that are reproducibly recovered from CRAC control experiments [32,48] are indicated with “H.” Additional supporting data are provided in S2 Data. (TIF) [file pbio.1002083.s011.tif]

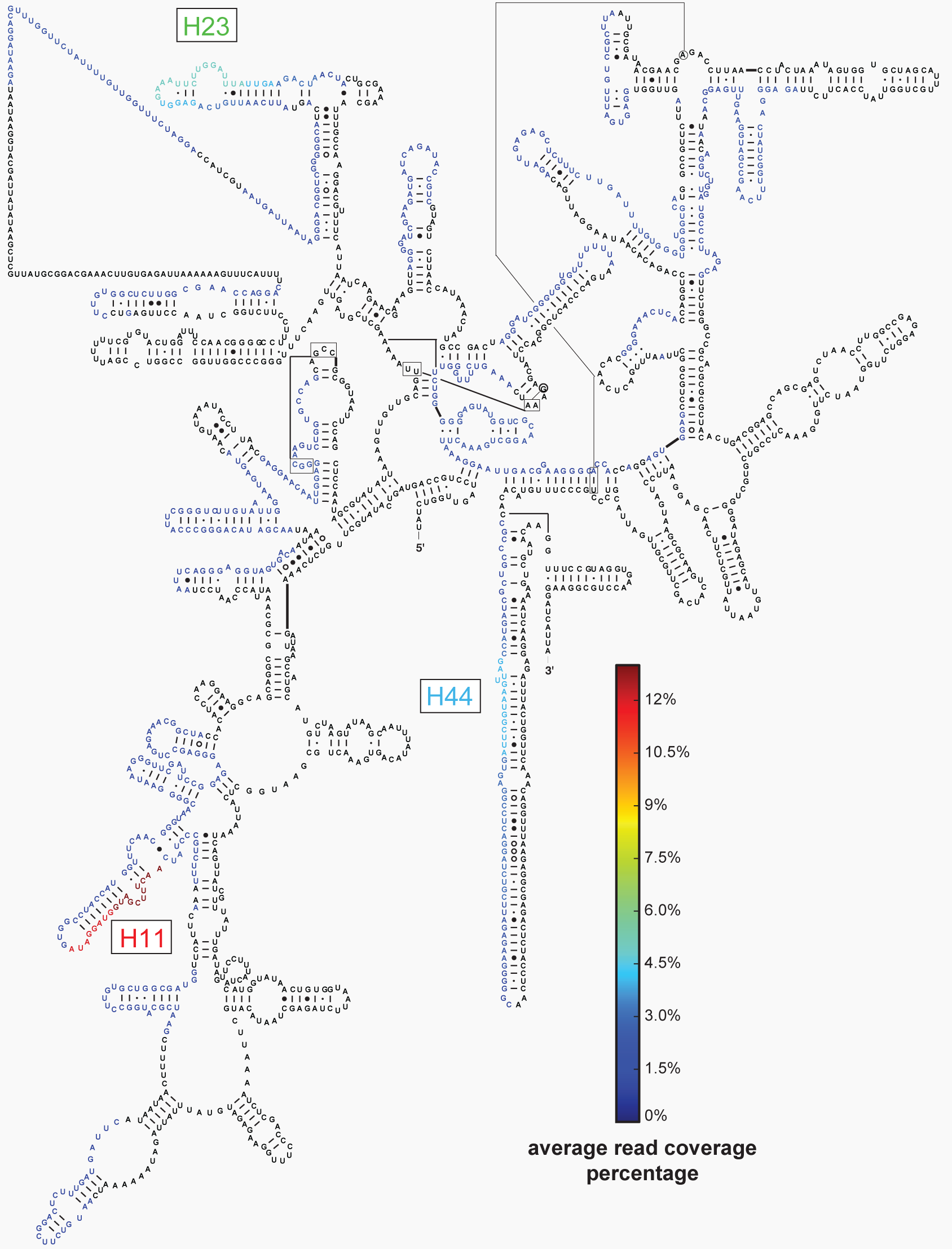

Supplement: S5 Fig — Shown is a secondary structure model for the S. cerevisiae 18S rRNA (available at http://www.rna.ccbb.utexas.edu). Colors indicate the average percentage of read coverage (n = 2) for each nucleotide in the 18S rRNA. Helices where consistently high cross-linking signals were observed are indicated with “H.” Additional supporting data are provided in S2 Data. (TIF) [file pbio.1002083.s012.tif]

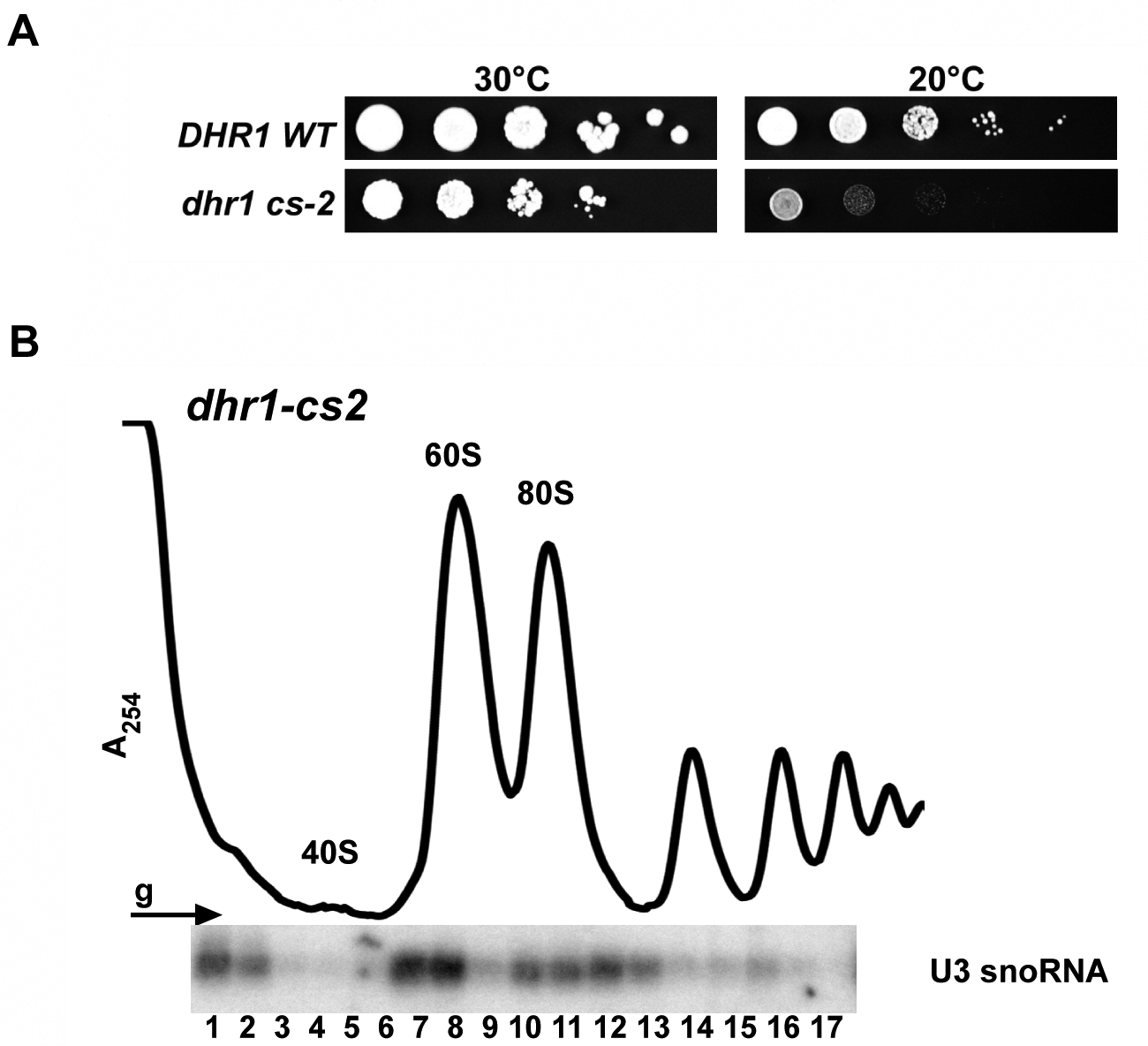

Supplement: S6 Fig — (A) 10-fold serial dilutions of AJY3715 (dhr1∆::KanMX) containing pAJ2593 (DHR1-WT) or pAJ2388 (dhr1-cs2) were spotted onto Ura-medium and incubated for 4 days at 20°C or 30°C. (B) Strain AJY3711 carrying plasmid pAJ2388 was cultured in SD Ura- galactose at 30°C. Glucose was added to 2% final concentration and cells were cultured for an additional 6 h at 20°C. Extracts were prepared and fractionated through 7%–47% sucrose gradients, RNA isolated, and Northern blotting for U3 were as described for Fig. 2. (TIF) [file pbio.1002083.s013.tif]

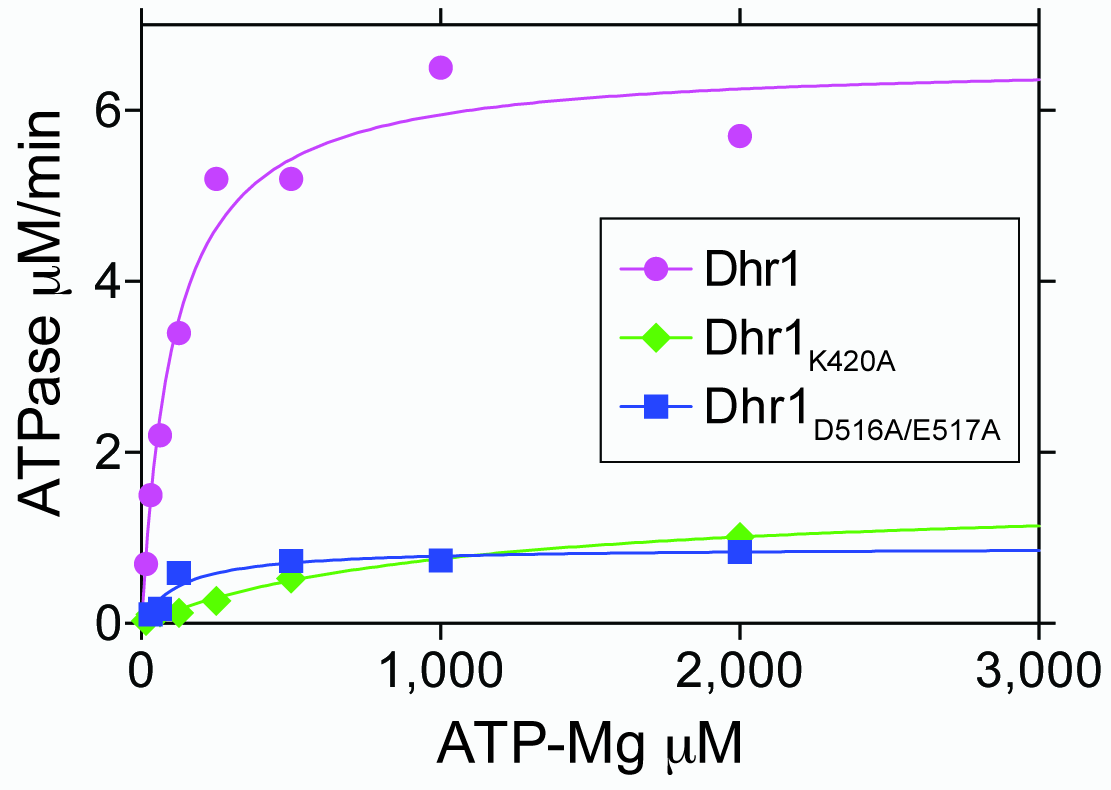

Supplement: S7 Fig — Initial velocities of Pi released after addition of ATP at RT in the presence of poly(A). ATP hydrolysis activity is plotted as a function of ATP concentration with either Dhr1 (purple), Dhr1D516A/E517A (blue), or Dhr1K420A (green). Additional supporting data are provided in S3 and S4 Data. (TIF) [file pbio.1002083.s014.tif]

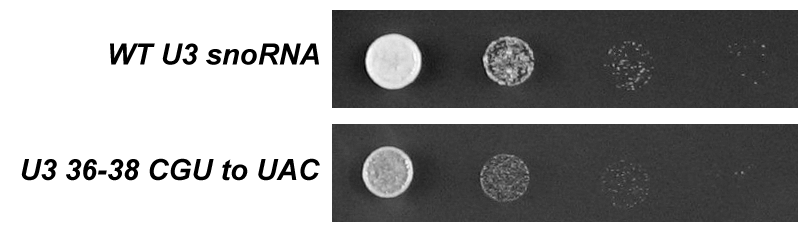

Supplement: S8 Fig — Nucleotides CGU at position 36 to 38 of U3 were mutated to UAC in pAJ2587. WT and mutant U3 were expressed in AJY3752 (P GAL-DHR1 P GAL-SNR17A snr17B∆) containing pAJ3095 (dhr1-cs2) and the ability of mutant U3 to suppress the cold-sensitive growth of dhr1-cs2 was assayed by serial dilution on His- glucose medium. Plates were incubated at 20°C for 6 days. (TIF) [file pbio.1002083.s015.tif]
