# Supplementary material for: The DEAH-box Helicase Dhr1 Dissociates U3 from the Pre-rRNA to Promote Formation of the Central Pseudoknot
Source: PLoS Biol. 2015 Feb 24;13(2):e1002083. doi: 10.1371/journal.pbio.1002083 (PMC4340053; doi:10.1371/journal.pbio.1002083)
Supplement: S1 Table — Mass spectrometric analysis was carried out on affinity purified Dhr1K420A (TEV-13xmyc-tagged) and mock (untagged) particles. Affinity purified particles were subjected to in-gel trypsin digestion and peptides identified by mass spectrometry. The number of peptide spectral matches (Σ #PSM) and molecular weight (MW) are given. Hits are grouped into known complexes and color-coded. As a rough proxy for abundance, PSM was divided by molecular mass and normalized to the value for Dhr1 (PSM/MW). (DOCX) [file pbio.1002083.s016.docx]

**Table S1**

|  | Σ# PSMs |  |  |  |  |
| --- | --- | --- | --- | --- | --- |
| Description | tagged | mock | MW [kDa] | Normalized  PSMs/MW | Complex |
| DHR1 | 176 | 0 | 144.9 | 1.00 | Dhr1 |
| MPP10 | 46 | 0 | 66.9 | 0.57 | Mpp10 |
| IMP3 | 9 | 0 | 21.9 | 0.34 |  |
| IMP4 | 15 | 0 | 33.5 | 0.37 |  |
| UTP4 | 27 | 0 | 87.7 | 0.25 | UtpA |
| UTP5 | 21 | 0 | 72.0 | 0.24 |  |
| UTP8 | 29 | 0 | 80.1 | 0.30 |  |
| UTP9 | 17 | 0 | 65.2 | 0.21 |  |
| UTP10 | 101 | 0 | 200.0 | 0.42 |  |
| UTP15 | 17 | 0 | 57.7 | 0.24 |  |
| UTP17 | 50 | 0 | 101.2 | 0.41 |  |
| PWP2 | 45 | 0 | 103.9 | 0.36 | UtpB |
| UTP6 | 23 | 0 | 52.4 | 0.36 |  |
| UTP12 | 44 | 0 | 106.3 | 0.34 |  |
| UTP13 | 29 | 0 | 91.0 | 0.26 |  |
| UTP18 | 8 | 0 | 66.4 | 0.10 |  |
| UTP21 | 31 | 0 | 104.7 | 0.24 |  |
| RRP7 | 2 | 0 | 34.4 | 0.05 | UtpC |
| UTP22 | 78 | 0 | 140.4 | 0.46 |  |
| NOP1 | 13 | 0 | 34.4 | 0.31 | Box C.D |
| NOP56 | 13 | 0 | 56.8 | 0.19 |  |
| NOP58 | 32 | 0 | 56.9 | 0.46 |  |
| RRP9 | 30 | 0 | 65.0 | 0.38 | U3 snoRNP |
| MTR4 | 55 | 0 | 122.0 | 0.37 | Exosome |
| CSL4 | 6 | 0 | 31.6 | 0.16 |  |
| RRP4 | 5 | 0 | 39.4 | 0.10 |  |
| RRP6 | 28 | 0 | 84.0 | 0.27 |  |
| RRP40 | 2 | 0 | 26.5 | 0.06 |  |
| RRP41 | 10 | 0 | 27.5 | 0.30 |  |
| RRP43 | 4 | 0 | 44.0 | 0.07 |  |
| RRP44 | 48 | 0 | 113.6 | 0.35 |  |
| RRP45 | 7 | 0 | 33.9 | 0.17 |  |
| RRP46 | 6 | 0 | 24.4 | 0.20 |  |
| RPS1A | 15 | 0 | 28.7 | 0.43 | 40S |
| RPS1B | 18 | 0 | 28.8 | 0.51 |  |
| RPS4A | 10 | 0 | 29.4 | 0.28 |  |
| RPS5 | 7 | 0 | 25.0 | 0.23 |  |
| RPS6A | 9 | 0 | 27.0 | 0.27 |  |
| RPS7A | 12 | 0 | 21.6 | 0.46 |  |
| RPS7B | 9 | 0 | 21.6 | 0.34 |  |
| RPS8A | 10 | 0 | 22.5 | 0.37 |  |
| RPS9B | 14 | 0 | 22.3 | 0.52 |  |
| RPS11A | 4 | 0 | 17.7 | 0.19 |  |
| RPS13 | 13 | 0 | 17.0 | 0.63 |  |
| RPS14A | 8 | 0 | 14.5 | 0.45 |  |
| RPS17A | 7 | 0 | 15.8 | 0.37 |  |
| RPS18A | 13 | 0 | 17.0 | 0.63 |  |
| RPS19A | 6 | 0 | 15.9 | 0.31 |  |
| RPS22A | 2 | 0 | 14.6 | 0.11 |  |
| RPS24A | 12 | 0 | 15.3 | 0.64 |  |
| RPS27A | 1 | 0 | 8.9 | 0.09 |  |
| RPS28B | 2 | 0 | 7.6 | 0.22 |  |
| RPS31 | 1 | 0 | 17.2 | 0.05 |  |
| RPS0A | 2 | 0 | 28.0 | 0.06 |  |
| UTP20 | 179 | 0 | 287.4 | 0.51 |  |
| RRP5 | 123 | 0 | 193.0 | 0.52 |  |
| RRP12 | 74 | 0 | 137.4 | 0.44 |  |
| BMS1 | 112 | 0 | 135.5 | 0.68 |  |
| UTP14 | 95 | 0 | 103.0 | 0.76 |  |
| UTP2/NOP14 | 43 | 0 | 94.2 | 0.38 |  |
| ENP1 | 40 | 0 | 55.1 | 0.60 |  |
| DIM1 | 34 | 0 | 35.9 | 0.78 |  |
| NEP1 | 33 | 0 | 27.9 | 0.97 |  |
| SAS10 | 33 | 0 | 70.2 | 0.39 |  |
| RCL1 | 25 | 0 | 40.1 | 0.51 |  |
| TRM1 | 22 | 0 | 62.1 | 0.29 |  |
| NOC4 | 22 | 0 | 63.6 | 0.28 |  |
| SOF1 | 21 | 0 | 56.8 | 0.30 |  |
| URA2 | 16 | 0 | 244.9 | 0.05 |  |
| MAK21 | 15 | 0 | 116.6 | 0.11 |  |
| UTP7 | 13 | 0 | 62.3 | 0.17 |  |
| ERB1 | 13 | 0 | 91.6 | 0.12 |  |
| EF1A | 11 | 0 | 50.0 | 0.18 |  |
| HAS1 | 10 | 0 | 56.7 | 0.15 |  |
| EFT1 | 7 | 0 | 93.2 | 0.06 |  |
| PNO1 | 6 | 0 | 30.3 | 0.16 |  |
| KRE33 | 5 | 0 | 119.3 | 0.03 |  |
| FCF2 | 5 | 0 | 25.6 | 0.16 |  |
| SSB1 | 4 | 0 | 66.6 | 0.05 |  |
| NOP12 | 4 | 0 | 51.9 | 0.06 |  |
| RPP0 | 3 | 0 | 33.7 | 0.07 |  |
| RPL6B | 3 | 0 | 20.0 | 0.12 |  |
| NOC2 | 3 | 0 | 81.6 | 0.03 |  |
| BUD21 | 3 | 0 | 24.4 | 0.10 |  |
| HTA1 | 2 | 0 | 14.0 | 0.12 |  |
| RPL2A | 2 | 0 | 27.4 | 0.06 |  |
| RPL4A | 2 | 0 | 39.1 | 0.04 |  |
| YEF3 | 2 | 0 | 115.9 | 0.01 |  |
| RPL8A | 2 | 0 | 28.1 | 0.06 |  |
| UTP11 | 2 | 0 | 29.7 | 0.06 |  |
| RPF2 | 2 | 0 | 39.6 | 0.04 |  |
| NOP2 | 2 | 0 | 69.8 | 0.02 |  |
| AZF1 | 2 | 0 | 101.1 | 0.02 |  |
| NOP7 | 2 | 0 | 69.8 | 0.02 |  |
| PUF6 | 2 | 0 | 75.1 | 0.02 |  |
| FCF1 | 2 | 0 | 21.6 | 0.08 |  |
| NOB1 | 2 | 0 | 51.7 | 0.03 |  |
| YTM1 | 2 | 0 | 51.3 | 0.03 |  |
| SMT3 | 2 | 0 | 11.6 | 0.14 |  |
| CTF3 | 2 | 0 | 84.2 | 0.02 |  |
| CDC19 | 1 | 0 | 54.5 | 0.02 |  |
| PGK1 | 1 | 0 | 44.7 | 0.02 |  |
| MIS1 | 1 | 0 | 106.2 | 0.01 |  |
| NOP4 | 1 | 0 | 77.8 | 0.01 |  |
| MAK5 | 1 | 0 | 87.0 | 0.01 |  |
| YEL023C | 1 | 0 | 78.3 | 0.01 |  |
| YDR417C | 1 | 0 | 13.1 | 0.06 |  |
| TMN2 | 1 | 0 | 76.3 | 0.01 |  |
| YLH47 | 1 | 0 | 52.1 | 0.02 |  |
