# Supplementary material for: The DEAH-box Helicase Dhr1 Dissociates U3 from the Pre-rRNA to Promote Formation of the Central Pseudoknot
Source: PLoS Biol. 2015 Feb 24;13(2):e1002083. doi: 10.1371/journal.pbio.1002083 (PMC4340053; doi:10.1371/journal.pbio.1002083)
Supplement: S2 Table — (DOCX) [file pbio.1002083.s017.docx]

**Table S2**

| **Strains used in this work** | | |
| --- | --- | --- |
| **Strain** | ***Genotype*** | **Source** |
| AJY3324 | *MATa dhr1∆::KanMX his3Δ1 leu2Δ0 met15Δ0 ura3Δ0/pAJ2388* | This study |
| AJY3335 | *MATalpha KanMX-P_GAL_-3xHA-DHR1 MATa his3Δ1 leu2Δ0 lys2Δ0 ura3Δ0* | This study |
| AJY3583 | *MATa P_GAL_::SNR17A::URA3 snr17b∆::LEU2 his3Δ1 leu2Δ0 met15Δ0 ura3Δ0* | This study |
| AJY3711 | *MATa KanMX-P_GAL_-3xHA-DHR1 MATa his3Δ1 leu2Δ0 met15Δ0 ura3Δ0* | This study |
| AJY3715 | *MATa dhr1∆::KanMX his3Δ1 leu2Δ0 ura3∆0* | This study |
| AJY3752 | *MATa KanMX-P_GAL_-3HA-DHR1 P_GAL_-SNR17A-URA3 snr17b∆::LEU2* | This study |
| BY4741 | *MATa his3Δ1 leu2Δ0 met15Δ0 ura3Δ0* | Open Biosystems |
| BY4742 | *MATalpha his3Δ1 leu2Δ0 lys2Δ0 ura3Δ0* | Open Biosystems |
| YKW100 | *MATa u3a∆ UASGAL:SNR17A::URA3 snr17b∆::LEU2 ura3-52 his3-∆ leu2 lys2-801 amber trp1∆63* | ([Wehner et al., 2002](#_ENREF_5)) |
