# Supplementary material for: The DEAH-box Helicase Dhr1 Dissociates U3 from the Pre-rRNA to Promote Formation of the Central Pseudoknot
Source: PLoS Biol. 2015 Feb 24;13(2):e1002083. doi: 10.1371/journal.pbio.1002083 (PMC4340053; doi:10.1371/journal.pbio.1002083)
Supplement: S3 Table — (DOCX) [file pbio.1002083.s018.docx]

**Table S3**

| **Plasmids used in the study** | | |
| --- | --- | --- |
| **Plasmid** | **Description** | **Source** |
| pAJ2158 | 18S rDNA locus in pUC18 | This study |
| pAJ2311 | WT *DHR1*-13myc *LEU2* CEN ARS | This study |
| pAJ2312 | *DHR1*-6xHIS in pET-21a | This study |
| pAJ2388 | *dhr1 cs-2 URA3* CEN | This study |
| pAJ2396 | *dhr1_K420A_*-6xHIS in pET-21a | This study |
| pAJ2587 | *SNR17A* *HIS3* CEN | This study |
| pAJ2593 | WT *DHR1 URA3* CEN ARS | This study |
| pAJ3081 | *dhr1_K420A_*-13myc *LEU2* CEN ARS | This study |
| pAJ3082 | WT *DHR1* (untagged) *LEU2* CEN | This study |
| pAJ3095 | *dhr1-cs2* (untagged) Clonat CEN | This study |
| pAJ3257 | *dhr1_D516A/E517A_*-6xHIS in pET-21a | This study |
| pAJ3317 | *DHR1*-6xHIS *LEU2* CEN | This study |
