# Supplementary material for: The DEAH-box Helicase Dhr1 Dissociates U3 from the Pre-rRNA to Promote Formation of the Central Pseudoknot
Source: PLoS Biol. 2015 Feb 24;13(2):e1002083. doi: 10.1371/journal.pbio.1002083 (PMC4340053; doi:10.1371/journal.pbio.1002083)
Supplement: S4 Table — (DOCX) [file pbio.1002083.s019.docx]

**Table S4**

| **Oligonucleotides used in the study** | | |
| --- | --- | --- |
| **AJO** | **Sequence** | **Description** |
| 130 | TCTTGCCCAGTAAAAGCTCTCATGC | D-A2 |
| 603 | TGTTACCTCTGGGCCCCGATTG | A2-A3 |
| 1566 | GATCCTCGAGGCTGAGGGCTTATTCTTGCC | DHR1 upstream |
| 1567 | GGTCACTAGTGGCTCATCGTCACTATATTGG | DHR1 downstream |
| 1686 | TAGATTCAATTTCGGTTTCTC | U3 |
| 1849 | GTGAGTTTCCCCGTGTTGAGT | 18S nt 2187–2200 |
| 1850 | CCCACCTATTCCCTCTTGC | A0-A1 |
